# Novel 8-Methoxycoumarin-3-Carboxamides with potent anticancer activity against liver cancer via targeting caspase-3/7 and β-tubulin polymerization

**DOI:** 10.1186/s13065-023-01063-5

**Published:** 2023-12-02

**Authors:** Ahmad Alzamami, Eman M. Radwan, Eman Abo-Elabass, Mohammed El Behery, Hussah Abdullah Alshwyeh, Ebtesam Al-Olayan, Abdulmalik S. Altamimi, Nashwah G. M. Attallah, Najla Altwaijry, Mariusz Jaremko, Essa M. Saied

**Affiliations:** 1 Clinical Laboratory Science Department, College of Applied Medical Science, Shaqra University, AlQuwayiyah 11961, Sahqra, Saudi Arabia; 2 Chemistry Department (The Division of Organic Chemistry), Faculty of Science, Port-Said University, Port-Said, Egypt; 3 Chemistry Department (The Division of Biochemistry), Faculty of Science, Port-Said University, Port-Said, Egypt; 4 Department of Biology, College of Science, Imam Abdulrahman Bin Faisal University, 31441 Dammam, Saudi Arabia; 5 Basic & Applied Scientific Research Centre, Imam Abdulrahman Bin Faisal University, P.O. Box 1982, 31441 Dammam, Saudi Arabia; 6 Department of Zoology, College of Science, King Saud University, Riyadh, Saudi Arabia; 7 Department of Pharmaceutical Chemistry, College of Pharmacy, Prince Sattam Bin Abdulaziz University, PO Box 173, 11942 Alkharj, Saudi Arabia; 8 Egyptian Drug Authority EDA Previously NODCAR, Cairo, Egypt; 9 Department of Pharmaceutical Sciences, Princess Nourah bint Abdulrahman University, P.O. Box 84428, 11671 Riyadh, Saudi Arabia; 10 Division of Biological and Environmental Sciences and Engineering, Smart-Health Initiative and Red Sea Research Center, King Abdullah University of Science and Technology, P.O. Box 4700, 23955-6900 Thuwal, Saudi Arabia; 11 Chemistry Department, Faculty of Science, Suez Canal University, Ismailia, 41522 Egypt; 12 Institute for Chemistry, Humboldt-Universität zu Berlin, Brook-Taylor-Str. 2, 12489 Berlin, Germany

**Keywords:** Liver cancer, Coumarin analogues, Antiproliferative activity, Cell cycle arrest, Apoptosis, Flow cytometric analysis, Caspase-3/7, β-tubulin polymerization, Molecular docking

## Abstract

In the present study, we explored the potential of coumarin-based compounds, known for their potent anticancer properties, by designing and synthesizing a novel category of 8-methoxycoumarin-3-carboxamides. Our aim was to investigate their antiproliferative activity against liver cancer cells. Toward this, we developed a versatile synthetic approach to produce a series of 8-methoxycoumarin-3-carboxamide analogues with meticulous structural features. Assessment of their antiproliferative activity demonstrated their significant inhibitory effects on the growth of HepG2 cells, a widely studied liver cancer cell line. Among screened compounds, compound **5** exhibited the most potent antiproliferative activity among the screened compounds (IC_50_ = 0.9 µM), outperforming the anticancer drug staurosporine (IC_50_ = 8.4 µM), while showing minimal impact on normal cells. The flow cytometric analysis revealed that compound **5** induces cell cycle arrest during the G1/S phase and triggers apoptosis in HepG2 cells by increasing the percentage of cells arrested in the G2/M and pre-G1 phases. Annexin V-FITC/PI screening further supported the induction of apoptosis without significant necrosis. Further, compound **5** exhibited the ability to activate caspase3/7 protein and substantially inhibited β-tubulin polymerization activity in HepG2 cells. Finally, molecular modelling analysis further affirmed the high binding affinity of compound **5** toward the active cavity of β-tubulin protein, suggesting its mechanistic involvement. Collectively, our findings highlight the therapeutic potential of the presented class of coumarin analogues, especially compound **5**, as promising candidates for the development of effective anti-hepatocellular carcinoma agents.

**Supplementary Information:**

The online version contains supplementary material available at 10.1186/s13065-023-01063-5.

## Introduction

Cancer remains a substantial worldwide health concern, impacting a considerable number of individuals annually across diverse cancer types [1]. Liver cancer is a notable malignancy within the spectrum of cancer types, characterized by its elevated incidence rates and unfavorable prognostic outcomes [2]. Hepatocellular carcinoma (HCC), commonly referred to as liver cancer [3], is an oncological condition characterized by the initiation of malignant growth in the hepatic cells, with the potential to metastasize to various anatomical sites [4]. Liver cancer is a significant global public health concern [5]. Liver cancer ranks as the sixth most frequently diagnosed cancer [6] and the fourth primary contributor to cancer-related mortality on a global scale [7], as reported by the World Health Organization (WHO). Chronic infections of hepatitis B and C viruses are widely acknowledged as the principal risk factors for the development of liver cancer [8], constituting more than 80% of reported cases [9]. Furthermore, there are additional risk factors that should be considered, including diabetes [10], obesity [11], non-alcoholic fatty liver (NAFLD) [12], excessive alcohol consumption [13], exposure to aflatoxins (toxins produced by certain types of molds) [14], and specific genetic conditions such as Wilson's disease [15] and hemochromatosis [16]. The selection of treatment modalities for liver cancer is contingent upon the extent and progression of the ailment [17]. Potential treatment options for this condition encompass various medical interventions, including surgical procedures like liver resection or transplantation, radiation therapy, chemotherapy, targeted therapies, and immunotherapies [18]. The efficacy of treatment is contingent upon the stage of liver cancer during the point of diagnosis, as well as the accessibility of resources and expertise required to effectively manage this intricate ailment [19].

Caspases, a class of protease enzymes known as cysteine proteases, are present in cells as inactive zymogens and are responsible for carrying out the process of apoptosis [20], also referred to as programmed cell death. Caspases can be classified into three distinct categories [21]: initiator caspases (including caspase 2, 8, 9, and 10) [22], executioner caspases (comprising caspase 3, 6, and 7) [23], and inflammatory caspases (encompassing caspase 1, 4, 5, 11, and 12) [24]. These entities exhibit mutual activation and give rise to a series of biochemical events [25]. The process of cellular dismantling involves the cleavage of various structural and regulatory proteins by executioner caspases [26]. It is important to note that initiator caspases are the first to be activated in this process. The caspases that play a role in the process of apoptosis can be categorized into two groups [27]: initiator caspases (such as caspase-9 in mammals) and effector caspases (such as caspase-3 and caspase-7 in mammals). One of the most intriguing techniques in cancer treatment is the activation of caspase-3-mediated induced apoptosis, which leads to cytotoxicity [28]. Many scientists and researchers have synthesized and studied bioactive compounds, including quinazoline, coumarin, thiosemicarbazone, chalcone, and pyrimidines that induce caspase-3-mediated apoptosis and cytotoxicity for cancer treatment [29, 30].

Growing empirical evidence indicates that tubulin proteins play a significant role in the metastatic progression of cancer [31]. Over the course of the past ten years, various studies have identified several tubulin isotypes that show promise as prognostic markers [32]. These isotypes have been found to have a correlation with aggressive disease, increased metastatic potential, and a higher likelihood of metastatic relapse in patients [33]. Among these isotypes, β-tubulin expression has been the primary focus of investigation [34]. Clinical data has demonstrated a significant association between elevated levels of β-tubulin protein expression and the manifestation of aggressive clinical characteristics as well as unfavorable prognosis in various types of cancer, such as pancreatic, glioblastoma, gastric, ovarian, breast, colorectal, and prostate. In both gastric and gliomas cancers, the expression of β-tubulin has been observed to be correlated with the presence of high-grade malignancy [35]. Previous studies have documented the existence of functional associations between β-tubulin and metastasis in mouse models of lung and pancreatic cancer. The depletion of β-tubulin leads to a reduction in anchorage independent growth, a significant characteristic associated with the metastatic capacity of non-small cell lung cancer cells [36]. The suppression of β-tubulin leads to the upregulation of the adhesion-associated tumour suppressor Maspin. This upregulation inhibits the outgrowth of cell migration, tumour spheroids, and enhances the sensitivity of non-small cell lung cancer cells to anoikic. Inhibiting the expression of β-tubulin led to diminished growth of pancreatic cancer cells, along with a decrease in their capacity to generate tumors and metastasize to distant organs. In a study conducted by Xiao et al*.*, it was shown that β-tubulin plays a significant role in imparting brain metastatic capabilities to breast cancer cells through the regulation of various crucial signaling molecules that are involved in cell adhesion and the process of metastasis [37]. The downregulation of β-tubulin resulted in the modulation of β3-integrin expression, leading to a decrease in extracellular matrix attachment. The observed phenomenon was found to be correlated with a decrease in metastatic capacity, as well as an enhancement in survival rates in a model of brain metastasis [38]. To sum up, these findings indicate that tubulin isotypes, particularly β-tubulin, have a notable impact on enabling the dissemination of cancer cells and could potentially act as predictive markers for the advancement of neoplastic diseases and the prognosis of patients. However, the exact mechanisms by which β-tubulin controls the metastasis process remain to be fully understood.

In order to enhance the efficacy of drug discovery, a pragmatic approach involves commencing the process with natural bioactive substances derived from medicinal plants or alternative natural origins. Coumarins, which were initially extracted from melilot flowers and tonka beans, have been extensively explored for their therapeutical applications, encompassing anti-inflammatory [39], anticancer [40], antiviral [41], antimicrobial [42], antioxidant [43], and anticoagulant [44]. Warfarin is widely recognized as a prominent pharmaceutical agent derived from coumarin [45]. This medication is classified as an original anticoagulant that has received approval for its efficacy in reducing the likelihood of blood clot formation [46] and preventing strokes in individuals with atrial fibrillation and/or those who have undergone cardiac valve replacement surgery [47]. The introduction of structural alterations to the coumarin scaffold not only results in the development of novel anticoagulants, but also induces a shift in the biological properties of newly synthesized coumarin analogs towards antispasmodic, vasodilating, antiproliferative, antibiotic, and chemoprotective activities [48]. The anticancer potentials of derivatives of coumarins have been the subject of investigation. For instance, the efficacy of coumarins containing the hydrazide-hydrazone moiety has been assessed in relation to their activity against drug-resistant pancreatic carcinoma cells and various other types of cancer cells [49]. Molecular hybridization is a valuable drug design strategy that can enhance the inhibitory potency of coumarins, while also offering the potential to improve their pharmacodynamic and pharmacokinetic properties [50]. The process entails the amalgamation of two or more pharmacophores, with or without the inclusion of any connecting group(s). Previous studies have demonstrated that the incorporation of supplementary pharmacophores into coumarin has resulted in enhanced activity, effectiveness, toxicity profiles and oral bioavailability [51].

**Fig. 1:**
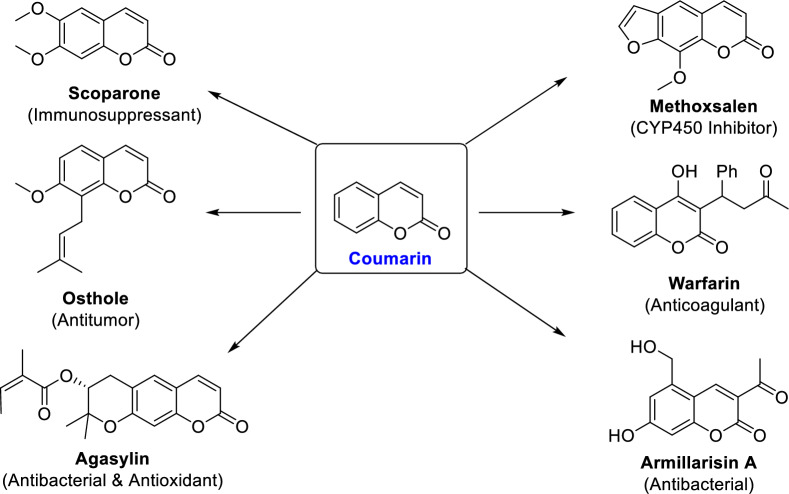
Representative Coumarin-based drugs with various pharmacological effects

**Fig. 2:**
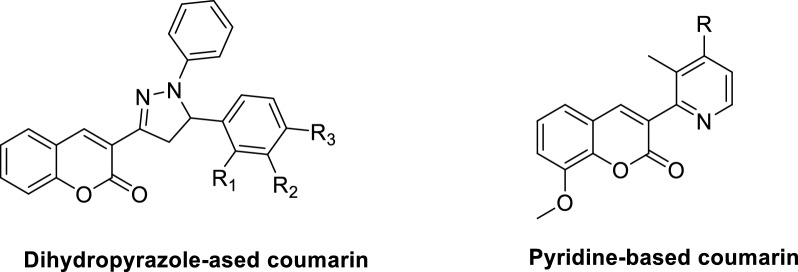
Representative sturctures of recently reported coumarin analogues with potent antitumor activity

The therapeutic applications of coumarin derivatives are contingent upon the specific characteristics and placement of substituents within the fundamental nucleus (Fig. 1). The impact of incorporating the dihydropyrazole moiety into the coumarin framework was examined in a study conducted by Hu et al*.* (Fig. 2) [52]. The authors demonstrated that this particular group of coumarin analogues exhibits significant hepatoprotective properties through the induction of apoptosis and the targeting of telomerase activity. The results of this investigation indicate that coumarin analogues exhibit promising therapeutic potential for the management of hepatic disorders. The study conducted by Fayed et al*.* demonstrated the diverse anti-cancer properties exhibited by coumarin analogues containing pyridine hybrids (Fig. 2). These compounds exhibited the ability to induce programmed cell death (apoptosis) and halt the progression of the cell cycle. Additionally, there was a notable increase in the activity of caspase-3, an enzyme involved in apoptosis [53].

The design and synthesis of coumarin hybrids represent a significant and novel approach within the realm of medicinal chemistry [54]. The coumarin core has demonstrated the ability to generate a diverse array of compounds that exhibit therapeutic potential against a range of diseases, encompassing microbial infections, cancer, inflammatory conditions, and neurodegenerative disorders. In recent times, a variety of drugs that are based on coumarin, such as coumadin, acenocoumarol, dicoumarol, phenprocoumon, and novobiocin, have received approval from the FDA and are currently being utilized in medical practice [55]. Additionally, a number of compounds that contain coumarin are currently undergoing clinical trials. The coumarin-based hybrid compounds were categorized by many research groups based on their shared biological activities, to identify their potential therapeutic targets [56]. As a result of their wide range of applications, coumarin analogues have emerged as promising research targets for the pharmaceutical industry [57]. The coumarin scaffold's adaptability and simplicity of production make it a useful starting point for the design of new compounds with enhanced pharmacological characteristics [58].

**Fig. 3:**
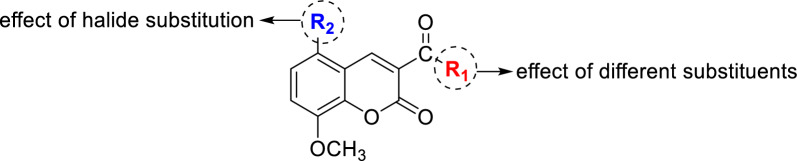
Illustrative diagram for structural features explored in the presented study

In light of the aforementioned and in our ongoing quest to discover new bioactive chemicals [59–68], we designed and synthesized a novel series of N-(substituted-phenyl)-8-methoxycoumarin-3-carboxamides. The design of these compounds was mainly based on exploring various structural features in the 8-methoxycoumarin scaffold (Fig. 3). The antiproliferative activity of designed compounds was extensively examined against HepG2 cells by conducting MTT assay and flow cytometric analysis. We further explored the mode of action of this class of coumarin analogue by assessing their potency toward β-tubulin polymerization and Caspase 3/7 proteins. Finally, we performed the detailed computational analysis to affirm the binding affinity of this class of compounds toward the targeted protein(s).

## Results and discussion

### Synthesis and characterization of novel 8-methoxycoumarine analogues

**Scheme 1:**
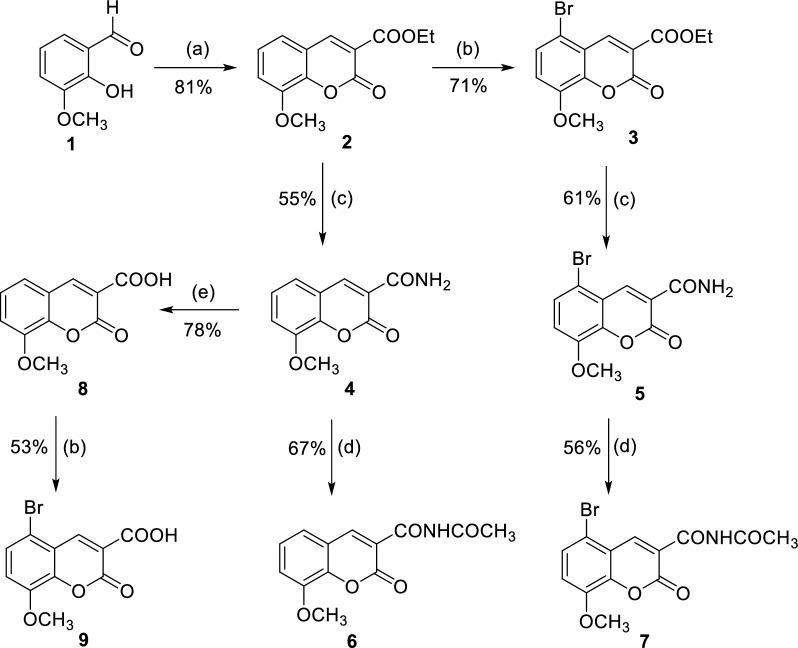
Synthesis of 8-methoxycoumarin-3-carboxamides (**4–7**) and 8-methoxycoumarin-3-carboxylic acid derivatives (**8,9**). Reagents and reaction conditions: **a** diethylmalonate, piperidine, fusion, 2 h; **b** Bromine, AcOH, 60 °C, 8 h; **c** Ammonium acetate, fusion, 6 h; **d** Acetic anhydride, reflux, 12 h; **e** acetic acid, 4N HCl, reflux, 16 h

The synthesis of 8-methoxycoumarin-3-carboxamides (4–7) and 8-methoxycoumarin-3-carboxylic acid derivatives (8,9) was successfully accomplished using a multi-step synthetic route, as shown in Scheme 1. Starting with 3-methoxy-2-hydroxybenzaldehyde (1) and diethyl malonate, ethyl 8-methoxycoumarin-3-carboxylate (2) was synthesized via a cyclocondensation reaction with piperidine as a base catalyst under fusion conditions. From ethyl 8-methoxycoumarin-3-carboxylate (2), ethyl 5-bromo-8-methoxycoumarin-3-carboxylate (3) was obtained with good efficiency (71% yield) through halogenation with bromine in glacial acetic acid. By subjecting ester coumarins 2 and 3 to ammonolysis with ammonia derived from ammonium acetate under fusion conditions, two key 8-methoxycoumarin-3-carboxamides were produced. Compound 4, 8-methoxycoumarin-3-carboxamide, was obtained in 53% yield, while compound 5, 5-bromo-8-methoxycoumarin-3-carboxamide, showed a slightly higher yield of 61%. The confirmation of the structures of 8-methoxycoumarin-3-carboxamide (4) and 5-bromo-8-methoxycoumarin-3-carboxamide (5) was achieved through further transformations. When subjected to an acetylation reaction, compound 5 was converted into 5-bromo-8-methoxycoumarin-3-carboxamide (7) with a satisfactory yield of 56%. Similarly, the acetylation of compound 4 led to the formation of compound (**6**), N-(acetyl)8-methoxycoumarin-3-carboxamide, with a yield of 61%. Furthermore, the reaction of 8-methoxycoumarin-3-carboxamide (**4**) with 4N hydrochloric acid in acetic acid under reflux conditions yielded 56% of 8-methoxycoumarin-3-carboxylic acid (**8**). Lastly, the synthesis of 5-bromo-8-methoxycoumarin-3-carboxylic acid (**9**) involved the halogenation of compound **8** using bromine in glacial acetic acid at a temperature of 60 °C. Overall, the study successfully demonstrated the synthesis of a diverse range of 8-methoxycoumarin-3-carboxamides and 8-methoxycoumarin-3-carboxylic acid derivatives using ethyl 8-methoxycoumarin-3-carboxylate as the starting material. The described synthetic pathway offers valuable insights into the potential applications of these compounds in various scientific disciplines, including medicinal chemistry.

The chemical identities and purities of compounds **4–9** were verified through the utilization of several spectroscopic techniques [69], namely proton nuclear magnetic resonance spectroscopy (^1^H NMR), carbon-13 nuclear magnetic resonance spectroscopy (^13^C NMR), and electron ionization mass spectrometry (EI-MS). These techniques collectively provided confirmation of the structural compositions of the compounds. The infrared spectra exhibited distinct absorption bands that corresponded to specific functional groups, including carbonyl, amide, and bromine [70]. These observations provided evidence for the existence of these functional groups within the synthesized compounds. The ^1^H-NMR spectra of compounds 4 and 5 exhibited a singlet signal at δ 3.94 ppm, which can be attributed to the presence of three protons originating from the methoxy (OCH_3_) group. The proton at position 4 of the coumarin ring in compounds 4 and 5 exhibited singlet signals at chemical shifts of δ 8.84 and 8.85 ppm, respectively. The protons associated with the NH_2_ group in compound 4 exhibit two singlet signals with chemical shifts of δ 7.95 and 8.09 ppm. Conversely, compound 5 displays a broad singlet signal at δ 8.09 ppm, indicating the presence of the NH_2_ group. In compound 4, the aromatic protons exhibit multiplet signals within the chemical shift range of δ 7.35–7.52 ppm.

In compound 5, two doublet signals are observed at δ 7.38 and 7.67 ppm, indicating the presence of two protons in the aromatic ring. The ^13^C-NMR spectra of compounds 4 and 5 displayed distinct signals at δ 162.98, 160.52, and 162.44, 159.80, respectively, which can be attributed to the presence of carbonyl groups in amides and coumarin rings. Additionally, the carbon signals corresponding to the methoxy groups in compounds 4 and 5 were observed at δ 3.94 ppm. The carbon signals corresponding to the C-O and C-4 positions of the coumarin ring in compounds 4 and 5 were detected at chemical shifts of δ 148.50, 146.72, 143.80 and 146.70, 141.91, 144.83 ppm, respectively. Additionally, the carbon signals in the aromatic region and the C-3 position of the pyranone ring were observed within the chemical shift range of δ 128.84–112.65 ppm. The mass spectra resulting from electron impact ionization of substances 4 and 5 exhibit ion peaks at *m/z* 219 and *m/z* 297, which correspond to the molecular formulas C_11_H_9_NO_4_ and C_11_H_8_BrNO_4_, respectively.

The absence of a proton signal at δ 8.09 ppm in the ^1^H-NMR spectrum of compound 7 indicates the absence of the amino (NH_2_) group. Additionally, the presence of two new singlet signals at δ 11.09 and 2.33 ppm suggests the presence of one proton in the NH group and three protons in the methyl function of the acetyl (COCH_3_) group. The ^13^C-NMR spectrum of compound 7 revealed the presence of two distinct carbon signals at chemical shifts of δ 171.71 and 25.59 ppm. These findings provide evidence for the formation of the acetyl derivative (7) as a result of the incorporation of the acetyl group (COCH_3_). The carbon signals corresponding to the remaining atoms in 8-methoxy-5-bromocoumarin-3-carboxamide are detected within the anticipated spectral regions. The verification of the structures of coumarin-3-carboxylic acid derivatives (8 and 9) was conducted through the analysis of the signals observed in the ^1^H-NMR spectra [71]. The spectrum of 8 demonstrated the lack of two proton signals attributed to the presence of the amino (NH_2_) group. The absence of these signals served as confirmation of the formation of an acid derivative. The protons located on the coumarin ring in compound 8 consist of H-4 of coumarin, as well as multiplet signals originating from the aromatic protons. ^1^H-NMR spectrum of compound 9 exhibited a singlet peak at a chemical shift of 8.45 parts per million (ppm), corresponding to the H-4 proton of the coumarin ring. Additionally, two doublet peaks were observed at chemical shifts of 7.59 and 7.30 ppm, which can be attributed to the two protons of the aromatic ring. The mass spectra analysis of compounds 8 and 9 revealed the presence of ion peaks at *m/z 220* and *m/z 298*, which can be attributed to the molecular formulas C_11_H_8_O_5_ and C_11_H_7_BrO_5_, respectively.

### Assessment of cytotoxic activity against HepG2 Cells

**Fig. 4:**
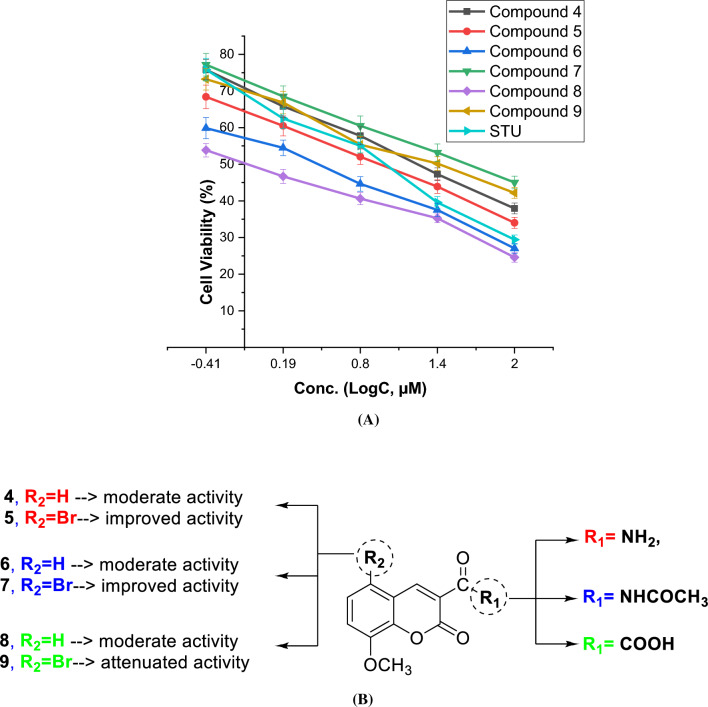
**A** The dose-dependent cytotoxic activity of synthesized compounds **4–9** toward HepG2 cells, as compared to staurosporine. The presented data shows the mean ± standard deviation of the mean obtained from at least three independent experiments. **B** Summary of presented structure–activity relationships showing the effect of different substituents

In our study, we investigated the impact of several newly synthesized coumarin derivatives (4–9) on the viability of HepG2 cells, a cell line derived from hepatocellular carcinoma. To assess the antitumor properties of these compounds, we utilized the 3-(4,5-dimethylthiazol-2-yl)-2,5-diphenyl tetrazolium bromide (MTT) colorimetric assay, a widely used method to evaluate cell viability and cytotoxicity. As a reference, we included staurosporine, a known anticancer compound, in our evaluation. The HepG2 cell line was exposed to various concentrations of the coumarin derivatives, and the dose-dependent cytotoxicity was assessed (Additional file 1: Table S1). The obtained IC_50_ values, which represent the concentration at which 50% of cell viability is inhibited, are presented in Table 1 and Fig. 4. Our results demonstrated that the newly synthesized coumarin derivatives exhibited a diverse range of antitumor effects, ranging from moderate to highly effective. Among the tested compounds, the main scaffold of 8-methoxycoumarin-3-carboxamide (**compound 4**) showed moderate antiproliferative activity with an IC_50_ of 17 µM. Interestingly, the introduction of an acetyl group to the 3-carboxamide of compound 4 (compound 6) resulted in improved cytotoxic activity with an IC_50_ of 2.3 µM. This suggests that the newly introduced acetyl group may play a crucial role in the binding of the compound to its targeted protein(s) by providing a site for hydrogen bonding acceptors. Moreover, the bromination of 8-methoxycoumarin-3-carboxamide at position-5 (compound 5) led to a significant increase in cytotoxic activity with an IC_50_ of 0.9 µM. This indicates that the bromination of the coumarin moiety at position-5 contributes to enhancing the cytotoxicity of the compound. Conversely, the acetylation of the 3-carboxamide moiety of compound 5 (compound 7) resulted in a considerable attenuation of cytotoxic activity with an IC_50_ of 2.3 µM. This suggests that the presence of both acetyl and bromo groups in the coumarin scaffold might lead to steric hindrance, affecting the compound's cytotoxic potential. Furthermore, the hydrolysis of 8-methoxycoumarin-3-carboxamide (compound 4) to the corresponding 3-carboxylic acid (compound 8) demonstrated a significant improvement in antiproliferative activity with an IC_50_ of 5 µM. This indicates the importance of the hydrogen bonding acceptor group (C = O) at position-3 for the cytotoxicity of this class of compounds. On the other hand, the bromination of 8-methoxycoumarin-3-carboxylic acid scaffold at position-5 (compound 9) led to an almost complete loss of cytotoxicity, with an IC_50_ of 41 µM. This suggests that the newly formed methoxycoumarin-3-carboxylic acid has a unique binding mode that differs from the corresponding 3-carboxamide analogue.

**Table 1:** Cytotoxic evaluation (IC_50_, µM) of compounds **4–9**, as compared to staurosporine, against human liver carcinoma HepG2 cells and WI 38 normal cells

Comp No	IC_50_ value (µM)	
	HepG2	WI 38
4	17.2 ± 1.05	NT^*^
5	0.9 ± 0.05	24.1 ± 1.46
6	23.6 ± 1.43	NT^*^
7	2.57 ± 0.16	NT^*^
8	5.18 ± 0.31	NT^*^
9	41.6 ± 2.52	NT^*^
STU	8.4 ± 0.51	26.7 ± 1.62

^*^^NTnot tested. The data provided represents the average value with its standard deviation of the mean (SEM), obtained from at least three separate experiments^

Among screened compounds, compound **5** displayed the most significant inhibitory effect, with an IC50 value of 0.9 µM. To put this into perspective, we also compared the activity of compound 5 with that of the anticancer drug staurosporine, which had an IC_50_ value of 8.4 µM. The substantial difference in IC_50_ values suggests that compound 5 possesses higher potency in suppressing the growth of HepG2 cells compared to the standard drug staurosporine. Encouraged by the promising results against liver cancer cells, we decided to investigate further and assess the antiproliferative activity of compound **5** against normal lung cells (WI-38 cells). It is essential to evaluate the selectivity of potential anticancer agents to ensure minimal harm to healthy cells. Our findings indicate that compound **5** exhibits a lower cytotoxic effect on WI38 cells compared to the anticancer drug staurosporine. The IC_50_ values for compound **5** against WI38 cells were 24.1 µM, while staurosporine had IC_50_ values of 26.7 µM (Additional file 1: Figure S9). This data demonstrates that compound **5** has a relatively higher selectivity for cancer cells over normal cells, making it a more attractive candidate for further investigation as an anticancer agent. Taken together, our study highlights the potential of the newly presented class of coumarin analogues, particularly compound **5**, as promising candidates for the development of novel anticancer drugs targeting liver cancer. The remarkable inhibitory activity of compound **5** against HepG2 cells, coupled with its reduced cytotoxicity towards normal lung cells, underscores its potential as a lead scaffold for the development of anticancer agents against liver cancer.

### Assessment of cell cycle analysis

**Fig. 5:**
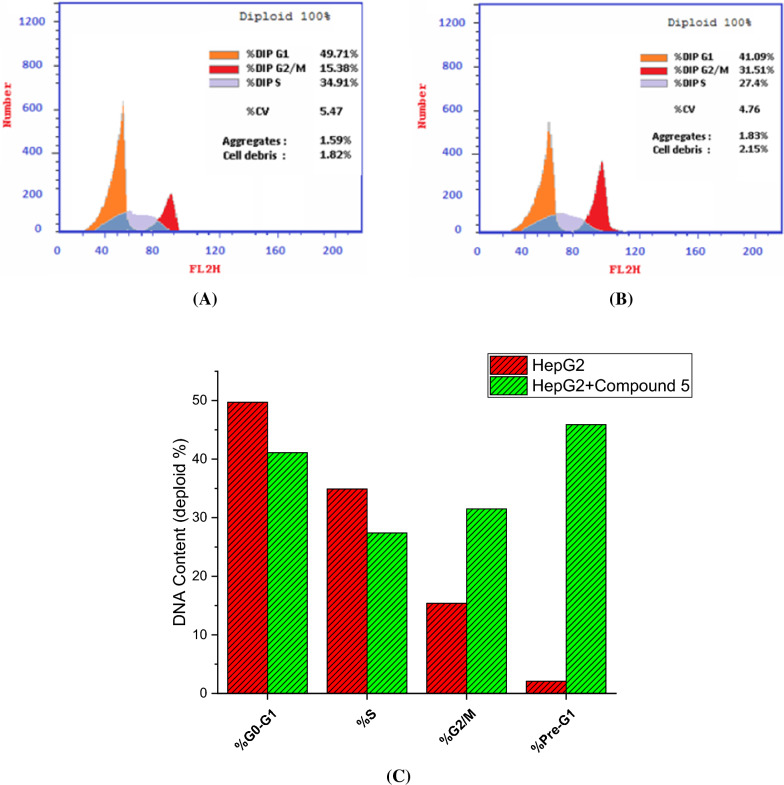
Flow cytometric analysis of the cell cycle phases in untreated HepG2 cells (**A**) and HepG2 cells treated with compound **5** (0.9 µM) (**B**). **C** DNA content in the different cell cycle phases of HepG2 cells

Next, our investigations focused on evaluating the effects of compound **5** on the cell cycle of HepG2 cells utilizing flow cytometric analysis [72]. The cells were treated with compound 5 at a concentration corresponding to the IC_50_ value and the cell cycle distribution was then analyzed, and the results were compared with untreated control cells. The findings depicted in Fig. 5 revealed significant alterations in the cell cycle distribution upon treatment with compound 5. The treatment led to a reduction in the cell population in the G0/G1 and S phases, with the percentage of cells in G0/G1 phase decreasing from 49.71% to 41.09%, and the percentage of cells in the S phase decreasing from 34.91% to 27.4%. Conversely, there was a notable increase in the cellular population at the G2/M and pre-G1 phases. The percentage of cells in the G2/M phase rose from 15.38% to 31.51%, while the percentage of cells in the pre-G1 phase increased dramatically from 2.08% to 45.88%. These findings indicate that compound **5** induces a notable arrest in cell growth during the G1/S phase. The flow cytometric analysis of HepG2 cells treated with compound **5** provided valuable insights into its effects on the cell cycle distribution. The observed reduction in the percentage of cells in the G0/G1 and S phases indicates that compound **5** induces a slowdown in cell growth during these phases. The G0/G1 phase is the resting phase, where cells prepare for DNA replication and cell division, while the S phase is where DNA replication occurs. The decrease in cell population in these phases suggests that compound **5** interferes with the normal progression of the cell cycle, possibly by inhibiting key regulatory proteins involved in cell cycle control. Moreover, the considerable increase in the cellular population at the G2/M phase suggests that compound **5** induces cell cycle arrest at the G2/M checkpoint. This is a critical control point where the cell ensures that DNA replication is complete and free from errors before entering mitosis. The observed accumulation of cells in the G2/M phase indicates that compound **5** halts cell cycle progression beyond this point, possibly due to its impact on proteins involved in G2/M transition or mitotic spindle formation. The most remarkable finding was the dramatic rise in the pre-G1 population upon treatment with compound **5**. The pre-G1 phase is associated with cells undergoing apoptosis, which is a programmed cell death process. The significant increase in cells in the pre-G1 phase suggests that compound **5** effectively induces apoptosis in HepG2 cells. This implies that the treatment with compound 5 leads to the activation of apoptotic pathways, causing the cells to undergo DNA fragmentation and degradation, ultimately leading to cell death. The ability of compound **5** to induce both G2/M arrest and apoptosis is intriguing and suggests a multifaceted mechanism of action. G2/M arrest prevents cells from progressing through the cell cycle, while apoptosis eliminates the damaged or aberrant cells. These combined effects may contribute to the compound's potent anti-cancer activity against HepG2 cells. Taken together the flow cytometric analysis revealed that compound **5** induces a notable arrest in cell growth during the G1/S phase and triggers apoptosis in HepG2 cells by increasing the percentage of cells arrested in the G2/M and pre-G1 phases. These findings suggest that compound **5** has significant anti-cancer potential and merits further investigation as a potential candidate for cancer therapy.

### Annexin V-FITC/ PI screening

**Fig. 6:**
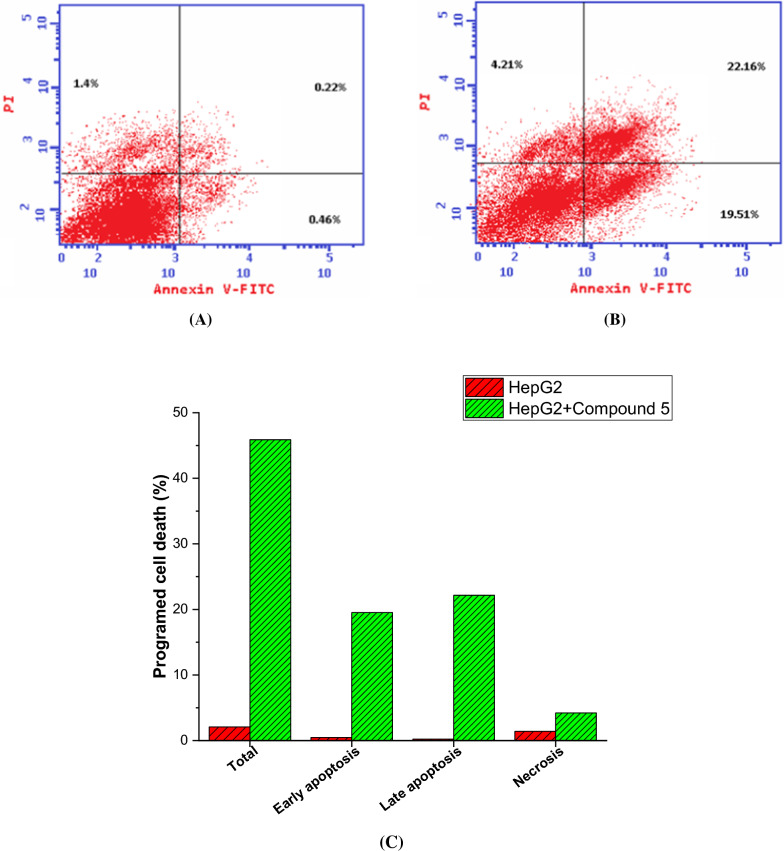
**A**: The apoptosis effect on human HepG2 cell line catalyzed by compound **5**. **B**: Compound **5** induced apoptosis effect in the human HepG2 cell line

Next, we have conducted Annexin V-FITC/PI screening to further investigate the impact of compound 5 on the growth of HepG2 cells and comprehensively analyze programmed cell death. The Annexin V-FITC/PI assay is a widely used method to differentiate between apoptotic and necrotic cells based on their membrane integrity and phospholipid exposure [73]. As shown in Fig. 6, the results indicated a substantial increase in the total percentage of programmed cell death in HepG2 cells upon treatment with compound 5. Specifically, the percentage of programmed cell death rose from 2.08% in untreated cells to 45.88% after treatment with compound 5, representing a remarkable 22-fold increase. Furthermore, the analysis revealed a notable increase in the percentage of apoptosis during both the early and late phases in compound 5-treated HepG2 cells. The percentage of cells undergoing early apoptosis increased from 0.46% in the control group to 19.51% after compound 5 treatment, indicating a significant 42-fold increase. Similarly, the percentage of cells in late-stage apoptosis rose from 0.22% in the control group to 22.16% after compound 5 treatment, representing an impressive 100-fold increase. On the other hand, compound 5 did not induce a remarkable effect on the necrosis pathway in HepG2 cells. The percentage of cells undergoing necrosis increased from 1.4% in untreated cells to 4.21% after compound 5 treatment, resulting in a threefold increase. The results from the Annexin V-FITC/PI screening provide crucial insights into the mode of action of compound 5 in inducing cell death in HepG2 cells. Annexin V-FITC is a marker that binds specifically to phosphatidylserine (PS), a phospholipid that becomes externalized on the cell membrane during early apoptosis. Propidium iodide (PI) is a dye that can penetrate cells with compromised membranes, typically found in late-stage apoptosis or necrosis. The substantial rise in the total percentage of programmed cell death upon treatment with compound 5 indicates that it effectively induces cell death in HepG2 cells. The significant increase in both early and late-phase apoptosis suggests that compound 5 triggers the apoptotic pathway at multiple stages, leading to cell demise. Notably, the lack of a significant effect on necrosis suggests that compound 5 primarily induces apoptosis rather than necrosis in HepG2 cells. This is important since apoptosis is a highly regulated process, and its induction is a favorable outcome for anti-cancer therapies, as opposed to necrosis, which is often associated with cell death caused by injury or external damage. Overall, these findings from the Annexin V-FITC/PI screening support the ability of compound 5 to induce apoptosis in HepG2 cells and its lack of significant induction of necrosis reinforce its potential as a promising candidate for anti-cancer therapy.

### Assessment of Caspase-3/7 activity

**Fig. 7:**
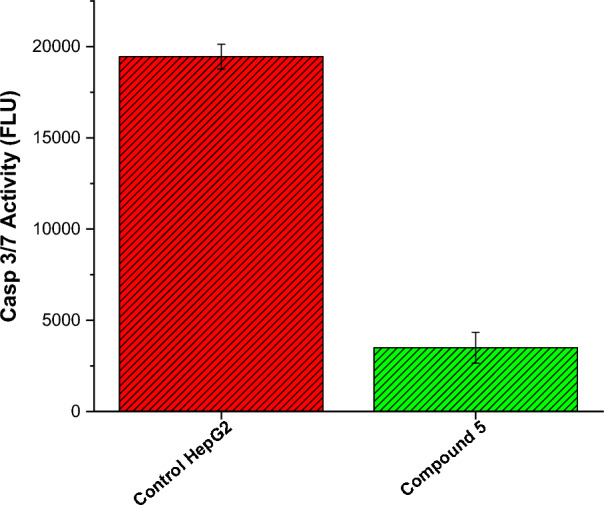
Effect of compound **5** on the level of caspase 3/7 in HepG2 cells

Caspase-3 is a cysteine-aspartic protease protein that facilitates the enzymatic cleavage of specific target proteins. It consists of two subunits, specifically a 12-kDa subunit and a 17-kDa subunit, which are characterized by the presence of three and five thiol functions, respectively [20, 74]. Caspases are initially produced as inactive zymogens, known as procaspases [75], which subsequently activated in response to specific internal and/or external signals, such as the increased production of reactive oxygen species (ROS). The crucial involvement of the terminal caspase-3/7 activation is observed in the initiation of apoptosis and subsequent cell demise in neoplastic cells [28]. To gain more insight into the apoptotic mode of action for compound **5**, we assessed the active caspase-3/7 levels in HepG2 cells utilizing a green flow cytometric assay. The results demonstrated that compound 5 treatment led to a substantial increase in the levels of active Caspase-3/7 in HepG2 cells. Compared to the control group of untreated HepG2 cells, there was a remarkable 5.6-fold rise in the levels of active Caspase-3/7 upon treatment with compound 5. This noteworthy increase in Caspase-3/7 activity indicates that compound 5 has the ability to promote apoptosis by triggering the activation of these key apoptotic proteins in HepG2 cells (Fig. 7). These findings provide valuable insights into the potential mechanism of action of compound 5 as an anti-cancer agent. Activation of Caspase-3/7 is a pivotal step in the apoptotic pathway, leading to the programmed destruction of cancer cells. The significant increase in active Caspase-3/7 levels observed in HepG2 cells treated with compound 5 indicates that this compound has a potent ability to induce apoptosis in cancer cells. The observed rise in Caspase-3/7 activity complements our results, which showed compound 5's inhibitory effect on β-tubulin polymerization. By disrupting microtubule dynamics and simultaneously promoting Caspase-3/7 activation, compound 5 likely triggers multiple pathways that contribute to the inhibition of cancer cell growth and the induction of apoptosis. These results support the hypothesis that compound 5's anti-cancer activity in HepG2 cells could be attributed, at least in part, to its capacity to activate the apoptotic pathway.

### Assessment of β-tubulin polymerization activity

**Fig. 8:**
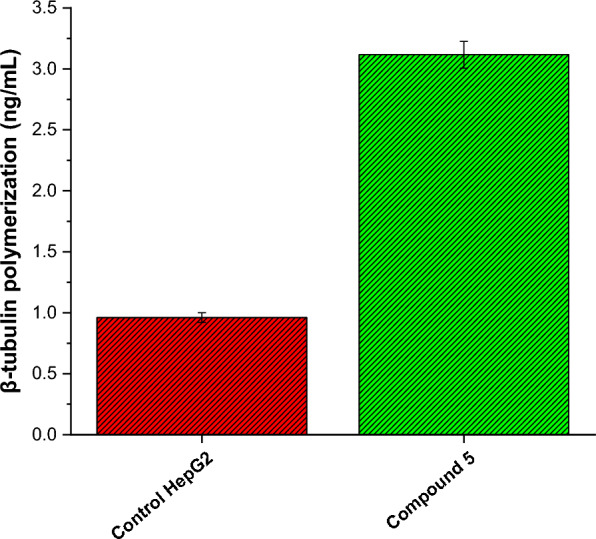
Inhibitory activity of compound **5** toward β-tubulin polymerization activity in HepG2 cells

Microtubules are widely recognized as a significant target for anti-cancer therapy due to their pivotal involvement in cellular division and the preservation of cellular morphology [76]. The impact of numerous anti-cancer agents is achieved through their ability to disrupt microtubule dynamics [77]. This disruption ultimately results in the deregulation of mitotic spindles, leading to cell cycle arrest in cancer cells and subsequently inducing apoptosis [78]. Toward this end, we further aimed to assess investigated the impact of compound **5** on β-tubulin polymerization activity in HepG2 cells. Thus, HepG2 cells were treated with compound 5 at (0.9 µM) for a duration of 24 h and the activity of β-tubulin polymerization was explored using spectrophotometry at 450 nm. The findings indicate that compound **5** exhibits a significant inhibitory effect toward β-tubulin polymerization as evidenced by a threefold reduction in the concentration of β-tubulin polymerization, as compared to the untreated HepG2 cells (Fig. 8). These findings indicate that compound **5** interferes with the process of microtubule assembly, thereby disrupting the proper formation of mitotic spindles. Microtubules are essential structures for cell division, and their dynamic behavior is critical for the accurate segregation of chromosomes during mitosis. By disrupting microtubule dynamics, compounds like **5** can induce mitotic defects and lead to cell cycle arrest in cancer cells, ultimately triggering programmed cell death (apoptosis). The observed inhibitory effect of compound **5** on β-tubulin polymerization strengthens the rationale for considering it as a candidate for anti-cancer therapy. By targeting the microtubule network, compound **5** may hinder the uncontrolled proliferation of cancer cells and potentially offer a more selective approach to cancer treatment. Overall, these results suggest that compound 5's anti-cancer activity may be attributed, at least in part, to its disruption of microtubule dynamics, leading to potential cell cycle arrest and apoptosis.

### In silico molecular modeling study

**Fig. 9:**
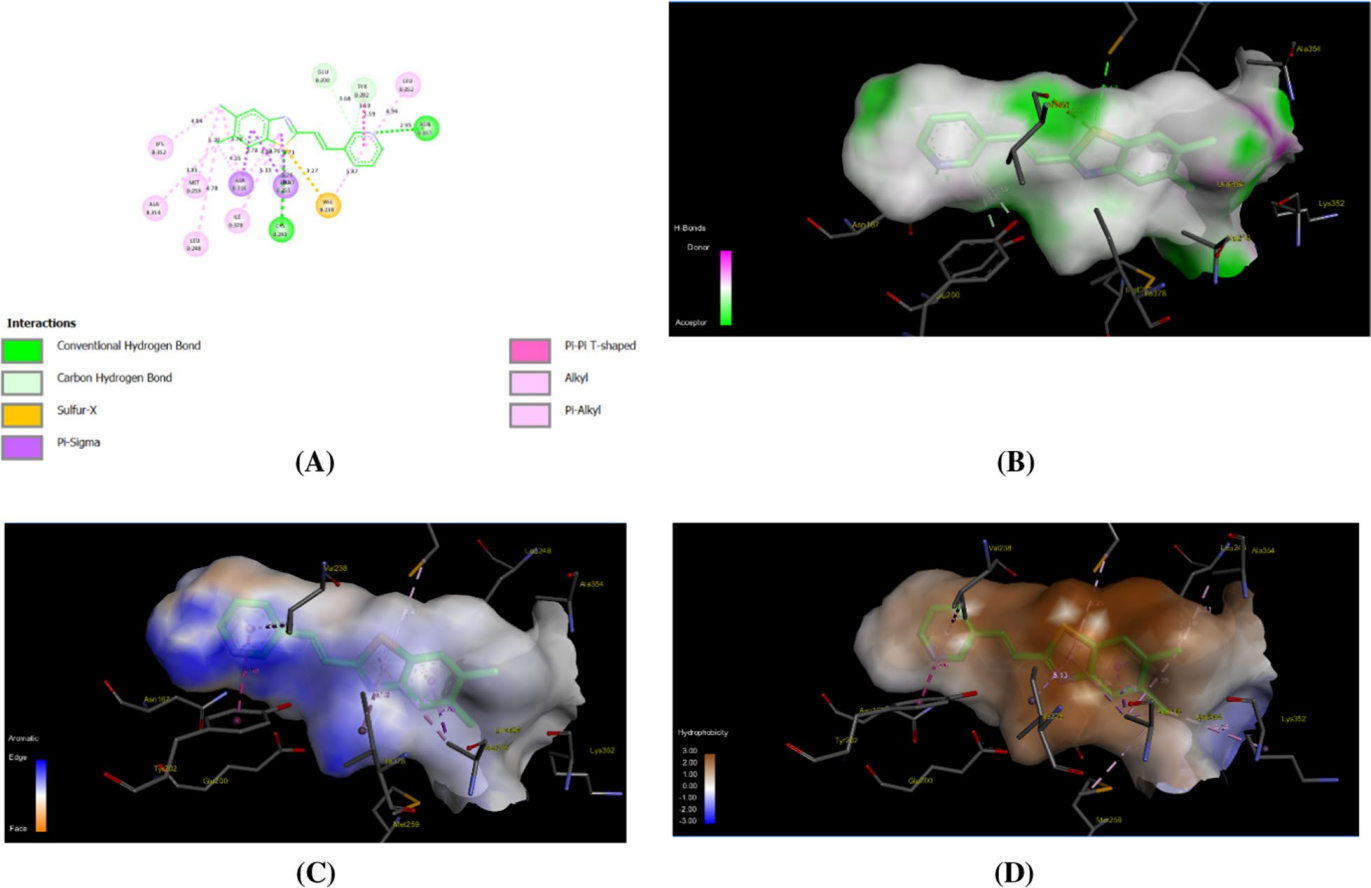
Representative binding modes of C2-ligand inside the active site of tubulin (PDB code: *4yh2*) protein through 2D (**A**), H-binding (**B**), aromatic (**C**), and hydrophobic interactions (**D**)

Finally, we aimed to affirm the inhibitory activity of compound **5** toward β-tubulin activity by assessing its binding affinity toward the active site utilizing molecular modelling analysis. The application of molecular docking simulation has been widely utilized and demonstrated to be efficacious in examining the interaction between a bioactive ligand and the active site of a particular protein, as well as in assessing its binding score [79]. For this purpose, we retrieved the crystal structure of tubulin (PDB code: 4yh2) along with its co-crystallized ligand, C2-ligand, from the Protein Data Bank (PDB). Prior to molecular modeling, unnecessary chains, water molecules, and other irrelevant entities were removed to ensure the accuracy of the docking process. The docking methodology was carefully adjusted to achieve low RMSD values, ensuring a reliable representation of the interactions as predicted by the crystal structure. The analysis revealed a series of interactions, including hydrogen bonding and hydrophobic interactions, between the C2-ligand and multiple amino acid residues within the binding pocket. The binding score obtained for the interaction between the C2-ligand and tubulin was -12.46 kcal/mol, indicating a strong binding affinity. This negative binding score suggests a favorable interaction, signifying that the C2-ligand has the potential to bind effectively to the active site of the tubulin protein. The formation of hydrogen bonds and hydrophobic interactions with specific amino acid residues within the binding pocket further supports the credibility of the docking results. The network of hydrophobic interactions involving residues like LEU255, ALA316, ALA354, and TYR202 demonstrates the involvement of specific amino acids in reinforcing the binding between the C2-ligand and tubulin. In addition to the hydrogen bonding interactions, a network of hydrophobic interactions was identified between the C2-ligand and specific amino acid residues in the binding pocket. The residues involved in these hydrophobic interactions included LEU255, ALA316, ALA354, and TYR202, with varying distances between the ligand and the amino acids (Fig. 9, Table 2).

**Table 2:** Binding score and interactions of C2-ligand into the active cavity of β-tubulin protein

Binding score (kcal/mol)	Hydrophobic interactions	Hydrophilic interactions (Hydrogen bonding)	Distance (A)
−12.46		ASN167	2.95
		CYS241	3.47
		Q4ED504	3.68
		Q4ED504	3.58
	LEU255		3.7, 3.75
	ALA 316		3.78, 3.77, 4.35
	ALA354		3.8
	TYR202		5.58

**Fig. 10:**
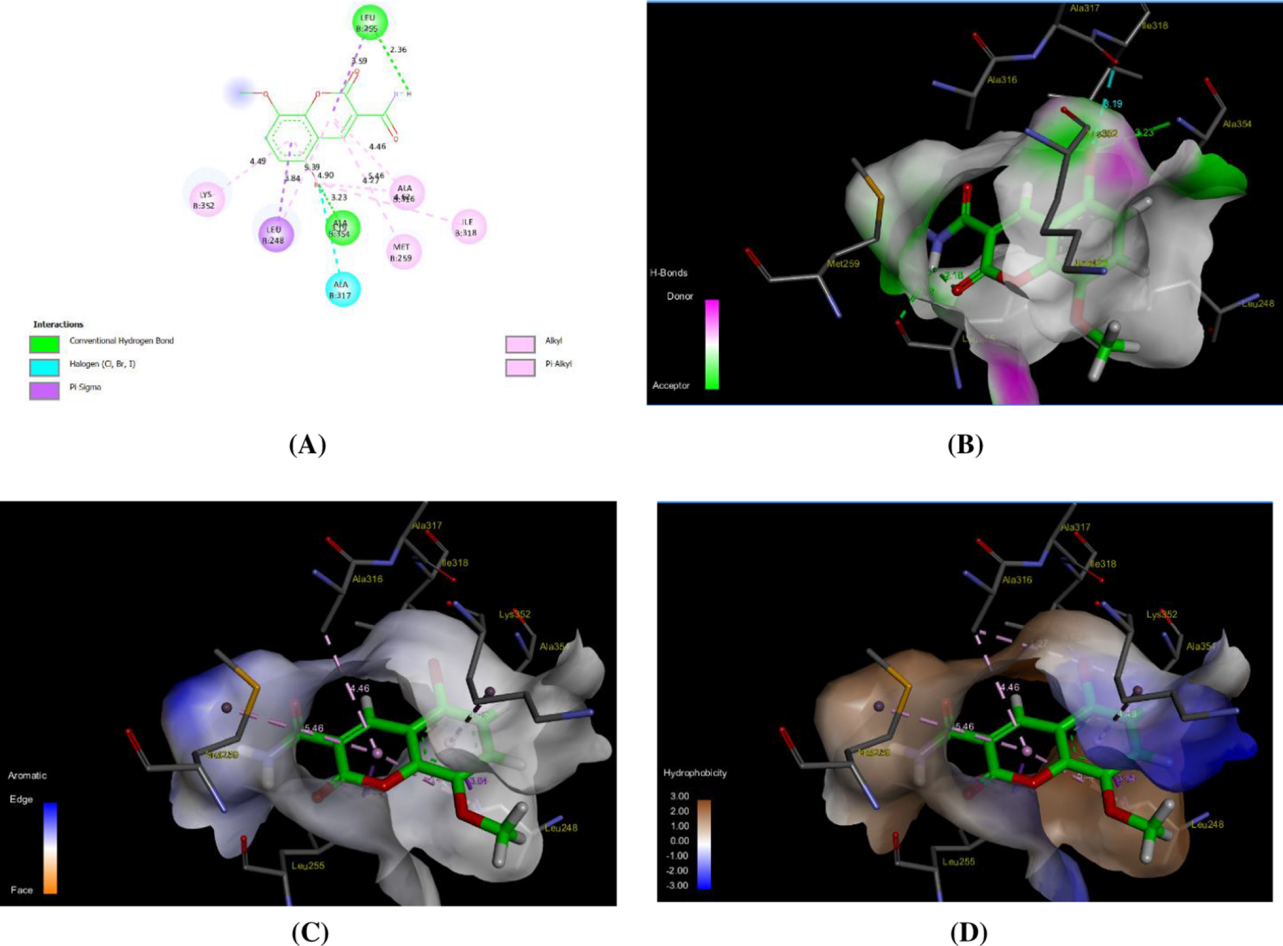
Representative binding modes of compound **5** inside the active site of tubulin (PDB code: *4yh2*) protein through 2D (**A**), H-binding (**B**), aromatic (**C**), and hydrophobic interactions (**D**)

In contrast to the C2-ligand, compound 5 demonstrated a notable affinity for binding to the active site of the tubulin protein, as indicated by a binding score of -11.89 kcal/mol. This score reflects a strong binding interaction between compound 5 and the tubulin protein, suggesting its potential as a tubulin inhibitor. The analysis of the binding mode revealed that compound 5 has the ability to interact with the tubulin protein through its 3-hydroxyphenyl-acetamide moiety, forming a total of four hydrogen bonds with various amino acid residues located within the binding pocket (Fig. 10). Hydrogen bonding interactions play a crucial role in stabilizing ligand–protein complexes and promoting specific binding. Furthermore, the binding affinity between compound 5 and its binding site was reinforced by a network of hydrophobic interactions involving specific amino acid residues within the cavity. The residues involved in these hydrophobic interactions included LEU248, LEU255, ALA316, ALA354, ILE318, LYS352, and MET259, with varying distances between the ligand and the amino acids (Table 3). These identified interactions provide valuable insights into the potential inhibitory activity of compound 5 against the tubulin protein. By binding to the active site of tubulin and forming hydrogen bonds and hydrophobic interactions, compound 5 may disrupt microtubule dynamics, a critical process in cellular division and maintaining cellular structure. This disruption could lead to cell cycle arrest and induce apoptosis, making compound 5 a promising candidate for anti-cancer therapy.

**Table 3:** Binding score and interactions of compound **5** into the active cavity of β-tubulin protein

Binding score (kcal/mol)	Hydrophobic interactions	Hydrophilic interactions (Hydrogen bonding)	Distance (A)
−11.89		ALA354	3.23
		LEU255	2.36, 2.18
	LEU248		3.84, 5.39
	LEU255		3.59
	ALA 316		4.27, 4.45
	ALA354		3.5, 4.9
	ILE318		4.62
	LYS352		4.49
	MET259		5.46

The current findings further support and validate our previous research concerning the inhibitory effect of compound 5 on the tubulin protein in an in vitro environment. Moreover, the results of our study suggest that the compound 8-methoxy-azacoumarin-3-carboxamide, a part of compound 5's chemical structure, holds potential as a foundational structure for the development of highly effective tubulin inhibitors. This information could inspire future drug design efforts to optimize compound 5 or its derivatives as potential anti-cancer agents targeting tubulin. Taken together, the molecular docking analysis demonstrates a notable affinity of compound 5 for the active site of the tubulin protein. The formation of hydrogen bonds and hydrophobic interactions supports its potential as a tubulin inhibitor. These findings contribute to our understanding of compound 5's inhibitory effect on tubulin and highlight its potential as a candidate for further development in the quest for novel and effective tubulin inhibitors for cancer treatment.

## Material and method

### Instrumentation, reagents and analysis

Reputable commercial hubs were the source of all the chemicals and solvents, used in the current study. During purchase of chemicals high level purity and basic standards of analytical grade were observed using different quality control protocols to ensure the precision and reproducibility of the experimental work. Reliable and accurate results during characterization and analysis of compounds was made sure by proper calibration of instruments, careful sample preparation and appropriate standerds used. Bruker avance 400 MHz and 100 MHz spectrometers were used for 1H and 13C NMRs respectively, while DMSO-d6 having tetra methyl silane solvent was used as internal standard. δ shifts (parts per million) is the key indicator while characterization and structural orientation of compounds while using the NMR. Microanalytical analysis for the determination of C, H, and N contents were carried out using Perkin-Elmer 2400 series with CHN analyzer, helping the accurate analysis and molecular formula determination of the compounds. EI-MS was performed using an Agilent Technologies 6890N gas chromatograph (GC) equipped with a 5973-mass spectrometer selective detector. The EI-MS analysis confirmed the molecular weights and ionic charges of the compounds by providing data on their mass-to-charge ratio. Crystalline compounds' melting points were determined without any corrections using an electrothermal melting point equipment. Purity, crystallinity, and identity of a compound is dependent on its melting point and for the verification of all above these mentioned parameters they were compared with standards. The vibrations and functional groups of compounds in the IR region were analyzed by recording their spectra using an infrared (IR) spectrometer (Bruker FT-8000). Infrared spectra provide information about the chemical bonds and functional groups present in a substance, allowing for more precise identification and characterization.

### Synthetic protocols and analytical assessments

#### Synthesis of Compound 2

Compound **1** was synthesized through the fusion of a mixture containing 0.01 mol of 3-methoxy-2-hydroxybenzaldehyde and 0.01 mol of diethylmalonate on a hot-plate. The reaction was conducted in the presence of 1 mL of piperidine for a duration of 5 min. Following that, a volume of 20 mL of ethanol was introduced into the reaction mixture, which was then subjected to reflux for a duration of 2 h. Following the completion of the reaction as judged by TLC analysis, the reaction mixture was subsequently cooled and carefully transferred into an ice-water bath with continuous stirring. Subsequently, the mixture was subjected to neutralization using a solution of (2%, aqueous) dil. hydrochloric acid. The resultant solid was subsequently gathered through the process of filtration. Ethyl 8-methoxycoumarin-3-carboxylate **(2)** was obtained according to above mentioned process, as white crystals. Yield 81%, m.p. 99 °C. IR (KBr) υ_max_: 1736, 1715 (C = O), 1605, 1585 (C = C), 1086, 1037 (C-O) cm^−1^. ^1^H-NMR (DMSO-d_6_, ppm) *δ*: 1.35 (t, 3H, CH_3_), 3.94 (s, 3H, OCH_3_), 4.33 (q, 2H, OCH_2_), 7.32–7.47 (m, 3H, Ar–H), 8.73 (s, 1H, H- coumarin). ^13^C-NMR (DMSO-d_6_, ppm) *δ*: 164.51, 163.10 (C = O), 157.23, 154.57 (C-O), 149.25 (C-coumarin), 135.03, 130.91, 125.42, 118.82, 118.33, 116.56 (C-coumarin and C-aromatic), 61.69 (OCH_2_), 56.60 (OCH_3_), 14.53 (CH_3_) (in agreement with previous reports [80, 81]).

#### Synthesis of brominated compounds 3 and 9

Compounds **1** and **8** (0.01 mol) were dissolved in 20 ml of glacial acetic acid. Subsequently, a solution of bromine (0.01 mol) in glacial acetic acid was added dropwise to the mixture of compounds **1** and **8** while stirring at a temperature of 60℃. After a duration of 10 min, the color of bromine was eliminated, and a yellow solution persisted. During this stage of the experiment, a solution containing 0.5–1.0 ml of Br_2_/AcOH was introduced with continuous agitation at ambient temperature for a duration of 8 h. Subsequently, the resulting mixture was carefully poured into water while maintaining a stirring motion. The solid that was generated underwent separation via filtration, followed by washing with water, drying, and subsequent recrystallization from an appropriate solvent, resulting in the formation of compounds **3** and **9**.

##### Ethyl 5-bromo-8-methoxycoumarin-3-carboxylate (3)

As pale-yellow crystals, yield 71%, m.p. 155 °C. IR (KBr) υ_max_: 1745, 1720 (C = O), 1610, 1588 (C = C), 1091, 1036 (C-O) cm^−1^. ^1^H-NMR (DMSO-d_6_, ppm) *δ*: 1.36 (t, 3H, CH_3_), 3.94 (s, 3H, OCH_3_), 4.34 (q, 2H, OCH_2_), 7.38 (d, 1H, Ar–H), 7.66 (d, 1H, Ar–H), 8.60 (s, 1H, H-coumarin ring). MS: *m/z* (%) = 328 (M^+^ + 2, 67.03), 326 (M^+^, 100), 300 (6.81), 299 (6.31), 283 (21.40), 281 (8.52), 255 (11.30), 254 (18.87), 248 (8.14), 247 (1.11), 219 (7.18), 181 (7.60), 156 (6.39), 119 (9.14), 105 (9.30), 104 (25.72). Anal. Calcd for C_13_H_11_BrO_5_ (M. wt. = 326): C, 47.85; H, 3.77. Found: C, 47.51; H, 3.20.

##### 5-Bromo-8-methoxycoumarin-3-carboxylic acid (9)

As colorless crystals, yield 53%, m.p. 210 °C. IR (KBr) υ_max_: 3360–2650 (br. OH), 1726–1718 (br. C = O), 1605, 1586 (C = C), 1081, 1031 (C-O) cm^−1^. ^1^H-NMR (DMSO-d_6_, ppm) *δ*: 3.94 (s, 3H, OCH_3_), 7.29–7.31 (d, 1H, Ar–H), 7.58–7.60 (d, 1H, Ar–H), 8.45 (s, 1H, H-coumarin). ^13^C-NMR (DMSO-d_6_, ppm) *δ*: 164.78, 156.72 (C = O), 146.61, 144.93 (C-O), 144.61 (C-coumarin), 128.40, 122.97, 118.34, 116.84, 112.24 (C-aromatic and C-3 of pyranone ring), 56.85 (OCH_3_). MS: *m/z* (%) = 300 (M^+^ + 2, 21.03), 299 (M^+^ + 1, 6.31), 298 (M^+^, 21.54), 297 (M^+^-1, 3.43), 256 (32.18), 255 (12.29), 254 (37.81), 228 (1.36), 227 (1.82), 226 (4.58), 213 (14.63), 212 (3.46), 211 (18.34), 204 (1.15), 203 (4.88), 185 (13.91), 183 (16.48), 175 (9.17), 157 (15.38), 156 (6.01), 155 (10.24), 141 (43.53), 140 (52.06), 139 (100), 120 (6.36), 119 (12.34), 113 (9.87), 111 (39.95), 104 (4.65), 103 (12.80), 96 (24.29), 95 (2.08), 94 (15.40), 93 (13.93), 91 (8.70), 89 (6.31), 76 (9.03), 75 (24.00), 74 (16.02). Anal. Calcd for C_11_H_7_BrNO_5_ (M. wt. = 298): C, 44.29; H, 2.35. Found: C, 44.04; H, 2.13.

#### Synthesis of 5-substituted 8-methoxycoumarin-3-carboxamides (4 and 5)

A blend comprising ester coumarin **2** or **3** (0.01 mol) and ammonium acetate (5 mol.) was subjected to fusion on a hot plate at a temperature range of 140℃ for a duration of 6 h. Subsequently, the mixture was cooled and introduced into water while being agitated. The precipitated powder was separated through the process of filtration, followed by washing with water, subsequent drying, and recrystallization using an appropriate solvent, resulting in the formation of compounds 4 and 5.

##### 8-Methoxycoumarin-3-carboxamide (4)

The entitled compound afforded as pale-yellow crystals. Yield 55%, m.p. 253 °C. IR (KBr) υ_max_: 3315–3187 (NH_2_), 1726–1690 (br. C = O), 1611, 1590 (C = C), 1083, 1045 (C-O) cm^−1^. ^1^H-NMR (DMSO-d_6_, ppm) *δ*: 3.94 (s, 3H, OCH_3_), 7.35–7.52 (m, 3H, Ar–H), 7.95 (s, 1H, NH_2_), 8.09 (s, 1H, NH_2_), 8.87 (s, 1H, H-coumarin). ^13^C-NMR (DMSO-d_6_, ppm) *δ*: 163.02, 160.62 (C = O), 148.49, 146.68, 143.79 (C-coumarin), 125.47, 121.58, 119.83, 119.51, 116.49 (C-aromatic and C-3 of pyranone ring), 56.71 (OCH_3_). MS: *m/z* (%) = 220 (M^+^ + 1, 14.50), 219 (M^+^, 100), 203 (35.33), 176 (11.18), 175 (10.51), 174 (6.21), 173 (5.06), 133 (32.07), 131 (3.02), 120 (7.96), 119 (14.36), 118 (12.96), 117 (3.92), 105 (29.26), 104 (9.60), 102 (5.94), 91 (12.96), 90 (11.88), 89 (31.65), 77 (45.22), 76 (35.40), 75 (16.45), 74 (16.85), 65 (10.09), 63 (22.72), 62 (17.43), 51 (23.99), 50 (18.49). Anal. Calcd for C_11_H_9_NO_4_ (M. wt. = 219): C, 60.27; H, 4.11; N, 6.39. Found: C, 60.12; H, 3.97, N, 6.11.

##### 5-Bromo-8-methoxycoumarin-3-carboxamide (5)

As pale yellow crystals, yield 61%, m.p. 285 °C. IR (KBr) υ_max_: 3336, 3189 (NH_2_), 1723–1691 (br. C = O), 1610, 1588 (C = C), 1067, 1035 (C-O) cm^−1^. ^1^H-NMR ((DMSO-d_6_, ppm) *δ*: 3.94 (s, 3H, OCH_3_), 7.37–7.39 (d, 1H, Ar–H), 7.67–7.69 (d, 1H, Ar–H), 8.09 (br. s, 2H, NH_2_), 8.76 (s, 1H, H-4 of coumarin) ppm. ^13^C-NMR (DMSO-d_6_, ppm) *δ*: 162.44, 159.80 (C = O), 146.81, 145.91 (C-O), 144.79 (C-coumarin), 128.84, 121.16, 118.63, 117.33, 112.65 (C-aromatic and C-3 of pyranone ring), 56.94 (OCH_3_). MS analysis: *m/z* (%) = 299 (M^+^ + 2, 74.90), 298 (M^+^ + 1, 27.75), 297 (M^+^, 100), 233 (8.13), 231 (14.27), 220 (4.09), 219 (53.00), 218 (76.14), 217 (2.89), 213 (13.40), 211 (18.13), 203 (19.43), 202 (19.56), 191 (1.09), 190 (49.77), 189 (3.06), 185 (6.91), 184 (6.25), 183 (10.63), 175 (7.45), 174 (12.27), 173 (4.95), 169 (3.59), 167 (2.57), 162 (2.12), 158 (7.69), 157 (12.25), 148 (6.07), 147 (9.90), 146 (2.79), 145 (2.76), 133 (8.56), 132 (6.32), 119 (22.87), 118 (16.41), 117 (14.48), 105 (12.75), 104 (14.84), 103 (44.53), 91 (20.07), 90 (15.95), 89 (25.89), 88 (9.37), 87 (13.04), 77 (12.09), 76 (29.94), 75 (46.82), 74 (20.96), 63 (7.39), 62 (2.34), 50 (3.37). Anal. Calcd for C_11_H_8_BrNO_4_ (M. wt. = 297): C, 44.48; H, 2.70; N, 4.70. Found: C, 44.18; H, 2.32, N, 4.47.

#### Synthesis of acetyl derivatives 6, 7

A mixture containing compound **4** or **5** (0.01 mol) dissolved in acetic anhydride (25 mL) was subjected to reflux for a duration of 12 h. Subsequently, the mixture was cooled and gradually added to a stirred solution of ice-water. The reaction mixture was allowed to stand for a duration of 24 h, following which the resultant product was obtained through the process of filtration. Subsequently, the product was subjected to a water wash and subsequently dried. Ultimately, the formation of compounds 6 and 7 was achieved through the crystallization of the solid product using an appropriate solvent.

##### N-acetyl 8-methoxycoumarin-3-carboxamide (6)

As colorless crystals, yield 67%, m.p. 285 °C. IR (KBr) υ_max_: 3227 (NH), 1725–1689 (br. C = O), 1605, 1582 (C = C), 1078, 1042 (C-O) cm^−1^. NMR spectra: no data because compound **6** insoluble in solvent. MS: *m/z* (%) = 262 (M^+^ + 1, 1.13), 261 (M^+^, 15.73), 220 (15.83), 219 (100), 204 (6.61), 203 (74.69), 202 (1.41), 191 (15.14), 190 (1.22), 176 (17.88), 175 (11.90), 174 (8.38), 173 (5.83), 161 (4.91), 148 (17.51), 147 (0.05), 146 (4.84), 133 (31.19), 132 (3.29), 120 (13.04), 119 (18.49), 118 (17.53), 117 (10.14), 105 (41.74), 104 (12.11), 103 (12.00), 102 (9.11), 92 (7.73), 91 (18.86), 90 (15.39), 89 (40.25), 88 (12.16), 87 (4.46), 78 (3.66), 77 (32.87), 76 (29.71), 75 (11.40), 74 (7.93), 73 (3.90), 65 (6.82), 63 (11.19), 62 (6.64), 51 (15.30), 50 (9.45). Anal. Calcd for C_13_H_11_NO_5_ (M. wt. = 261): C, 59.77; H, 4.21; N, 5.36. Found: C, 59.59; H, 4.01, N, 5.11.

##### N-acetyl 5-bromo-8-methoxycoumarin-3-carboxamide (7)

As pale yellow crystals, yield 56%, m.p. 220 °C. IR (KBr) υ_max_: 3225 (NH), 1723–1696 (br. C = O), 1612, 1590 (C = C), 1083, 1062 (C-O) cm^−1^. ^1^H-NMR (DMSO-d_6_, ppm) *δ*: 2.33 (s, 3H, COCH_3_), 3.94 (s, 3H, OCH_3_), 7.36–7.71 (m, 2H, Ar–H), 8.53 (s, 1H, H-4 of coumarin ring), 11.09 (br. s, 1H, NH). ^13^C-NMR ((DMSO-d_6_, ppm) *δ*: 171.71, 162.80, 162.41, 162.29, 159.78, 158.78 (C = O of more than two isomer), 146.77, 146.69, 145.90 (C-O), 144.81, 144.67, 144.60 (C-4 of pyranone ring), 129.13, 128.84, 122.96, 121.12, 118.61, 118.39, 117.69, 117.32, 112.65, 112.59 (C-aromatic and C-3 of pyranone ring), 57.00, 56.93 (OCH_3_ of two isomer), 25.59 (CH_3_). MS analysis: *m/z* (%) = 341 (M^+^ + 2, 27.20), 340 (M^+^ + 1, 9.59), 339 (M^+^, 39.51), 338 (M^+^-1, 20.28), 300 (10.38), 299 (45.60), 298 (15.98), 297 (39.20), 283 (49.54), 282 (48.53), 281 (54.54), 280 (55.45), 279 (8.65), 271 (23.90), 270 (10.78), 269 (27.81), 268 (28.07), 261 (6.48), 260 (100), 256 (21.67), 255 (9.86), 254 (26.83), 219 (16.45), 218 (46.59), 217 (71.35), 212 (20.94), 211 (15.71), 210 (25.44), 203 (21.80), 202 (36.00), 199 (27.55), 198 (19.40), 197 (24.21), 196 (22.98), 190 (41.75), 189 (19.40), 175 (24.61), 174 (15.69), 173 (15.51), 167 (15.07), 166 (19.41), 158 (16.49), 157 (24.22), 156 (14.27), 155 (28.27), 141 (39.93), 140 (25.11), 139 (83.88), 138 (82.81), 119 (19.60), 118 (16.56), 117 (12.17), 104 (6.87), 103 (35.59), 102 (15.79), 91 (8.05), 90 (7.08), 89 (11.82), 76 (6.27), 75 (22.37), 74 (11.19). Anal. Calcd for C_13_H_10_BrNO_5_ (M. wt. = 339): C, 46.02; H, 2.95; N, 4.13. Found: C, 45.87; H, 2.73, N, 4.02.

#### Synthesis of 8-methoxycoumarin-3-carboxylic acid (8)

A solution of coumarin-3-carboxamide (**4**, 0.01 mol) was dissolved in acetic acid (15 ml), then 15 ml 4N HCl was added. The reaction mixture was refluxed for 16 h, and then the contents of the reaction were poured into crushed ice. The resulting mixture was left at room temperature overnight, and the solid that formed was filtered off. The solid was then washed with water and dried. Afterward, recrystallization from ethanol was carried out to further purify the product. Colorless crystals. Yield 78%, m.p. 186 °C, IR (KBr) υ_max_: 3350–2851 (br. OH), 1725–1706 (br. C = O), 1606, 1591 (C = C), 1036, 1021 (C-O) cm^−1^. ^1^H-NMR (DMSO-d_6_, ppm) *δ*: 3.93 (s, 3H, OCH_3_), 7.31–7.46 (m, 3H, Ar–H), 8.73 (s, 1H, H-4 of coumarin). ^13^C-NMR (DMSO-d_6_, ppm) *δ*: 164.49, 156.90 (C = O), 148.95, 146.67 (C-O), 144.24 (C-4 of coumarin ring), 125.18, 121.52, 119.07, 118.96, 116.61 (C-aromatic and C-3 of pyranone), 56.60 (OCH_3_). MS analysis: *m/z* (%) = 221 (M^+^ + 1, 16.00), 220 (M^+^, 96.36), 203 (22.34), 177 (8.93), 176 (100.00), 161 (15.58), 149 (7.22), 148 (38.72), 147 (20.17), 146 (6.35), 141 (7.33), 139 (34.97), 133 (61.14), 131 (4.08), 120 (17.92), 119 (12.91), 118 (18.20), 117 (2.41), 111 (7.98), 105 (52.55), 104 (7.43), 103 (12.87), 102 (10.13), 91 (17.12), 90 (12.72), 89 (24.94), 88 (5.49), 77 (33.45), 76 (16.34), 75 (7.56), 65 (7.99), 63 (5.95), 62 (1.90), 51 (13.66). Anal. Calcd for C_11_H_8_O_5_ (M. wt. = 220): C, 60.00; H, 3.64. Found: C, 59.83; H, 3.33.

### In vitro assessment of cytotoxic activity against HepG2 and WI 38 cells

The antitumor efficacy of the recently developed coumarin compounds 4–9 was evaluated against the HepG2 and WI 38 cell lines (Sigma-Aldrich, USA, St. Louis, product number 85011430, and 90020107, respectively) utilizing the MTT assay technique. The cells were seeded in 96-well plates at a density of 1 × 10^4^ and incubated at 37 °C for 48 h in the presence of 5% CO_2_. Following the incubation period, the cells were subjected to various concentrations of the prepared molecules and subsequently incubated for a duration of 24 h. The MTT dye was introduced after a 24 h period of drug treatment and subsequently incubated for a duration of 4 h at a temperature of 37 °C. Subsequently, a volume of 100 μL of dimethyl sulfoxide (DMSO) was introduced into each well in order to facilitate the dissolution of the purple formazan that had been generated. The quantification of the color intensity of the formazan product, which serves as an indicator of the cellular growth condition, is performed by employing an ELISA plate reader set at a wavelength of 570 nm. The experimental conditions were implemented with a minimum of three replicates, and the experiments were conducted on at least three separate occasions.

### Cell cycle analysis

The quantification of DNA content in the cell cycle analysis was performed using a FACS Calibur flow cytometer at a wavelength of 488 nm, following the guidelines provided by the manufacturer. In this study, a total of 2 × 10^5^ cells per well were subjected to treatment with a specific compound, referred to as molecule 5, at concentrations corresponding to its IC_50_ for a duration of 24 h. Following the completion of the treatment, the cells underwent two rounds of washing and were subsequently resuspended in phosphate-buffered saline (PBS). Following the washing step, a volume of 0.7 ml of absolute ethanol was subsequently introduced, and the mixture was incubated at a temperature of -20 °C for a duration of 20 min. Following the washing step, a volume of 500 μl of RNase was introduced, and subsequently, the mixture was incubated for a duration of 30 min. Next, the addition of PI was carried out, followed by a 30-min incubation period, during which exposure to light was carefully avoided.

### Annexin V-FITC/ PI screening

Apoptosis detection in HepG-2 cells was conducted utilizing the BioVision® annexin‐V‐FITC apoptosis detection kit in accordance with the instructions provided by the manufacturer. The quantification of apoptosis was performed using a FACS Calibur flow cytometer, with a wavelength of 488 nm. In a concise manner, a total of 1–5 × 10^5^ cells were obtained through the process of centrifugation. The cells were subsequently exposed to compound 5 at concentrations corresponding to its IC_50_ value for a duration of 24 h. Following this treatment, the cells were resuspended in a binding buffer solution with a volume of 500 μl. The addition of Annexin-V-FITC and PI was performed. Subsequently, the specimens were subjected to incubation at ambient temperature for a duration of 5 min, while being shielded from light. The analysis of Annexin-V-FITC binding was conducted utilizing a dedicated signal detector.

### Assessment of caspase-3/7 activity

The HepG2 cell line was subjected to treatment with compound 5, and subsequently, an analysis was conducted using the Cell Event caspase-3/7 green flow assay. The Cytometry Assay Kit, identified by Catalog Number C10427 (Cayman, USA), is utilized in the field of academic research. The cell suspensions were subjected to centrifugation at 100 g for a duration of 7 min at a temperature of 37 °C, following which the supernatants were carefully extracted. The pellets were reconstituted in a 500 μl solution of phosphate buffer solution (PBS). Subsequently, a volume of 0.5 μL of the cell Event Caspase-3/7 Green detection Reagent was introduced to all cellular samples. The cells were subjected to incubation at a temperature of 37 °C for a duration of 30 min. Subsequently, the cells were assessed utilizing the BD FACS CALIBER flow cytometer.

### Assessment of β-tubulin polymerization activity

Dulbecco's Modified Eagle Medium (DMEM) (Invitrogen/Life Technologies) containing 10% fetal bovine serum (FBS) (Hyclone), 10 µg/mL of insulin (Sigma), and 1% penicillin–streptomycin was used for culturing the HepG2 cell line. The cancer cell suspension and the tested compound **5** were dispensed in a 96-well plate at a volume of 100 μL each per well. After incubating the plate for 18–24 h, the enzyme assay for tubulin was performed. The microtiter plate used in this study was pre-coated with an antibody specific to TUB β. Following that, standards or samples were added to their respective wells on the microtiter plate, along with a biotin-conjugated antibody that specifically binds to TUB β. Subsequently, the addition of Avidin-Horseradish Peroxidase (HRP) conjugate to individual microplate wells was followed by an incubation period. Upon addition of the TMB substrate solution, only the wells that contain TUB β, biotin-conjugated antibody, and enzyme-conjugated Avidin will manifest a discernible alteration in coloration. To conclude the enzymatic reaction between the enzyme and substrate, a solution of sulfuric acid was introduced, leading to a color change. This color change was then quantitatively measured using spectrophotometry at a specific wavelength of 450 nm ± 10 nm. To determine the concentration of TUB β in the samples, the optical density (O.D.) of the samples was compared to a standard curve. The experiments were conducted in triplicate, repeating the process three times for each sample, to ensure the accuracy and consistency of the results.

### In silico computational studies

The binding affinity of this particular chemical class to the active site of the tubulin protein was assessed through a thorough computational analysis using MOE software. The application of molecular docking enables the assessment of the binding affinity between small molecules and the binding site of a particular protein, thereby providing valuable insights into the mechanism of action of pharmacological compounds. Compound **5** was selected to investigate the affinity of the developed N-(substituted-phenyl)-8-methoxycoumarin-3-carboxamide analogues for the functional pocket of the tubulin protein. Using Chem.Draw, we were able to determine compound **5**'s 2-dimensional structure, which paved the way for additional computer investigation of the molecule. Tubulin protein crystallographic structures are abundant in the Protein Data Bank (PDB), which has greatly aided our research. We zeroed focused on the tubulin protein crystal structure (PDB code: *4yh2*) in particular. These structures allow for in-depth investigation of the proteins' structural characteristics and probable binding sites by providing information about their three-dimensional organization. Multiple experimental techniques were employed to prepare the 3D structures of the tubulin protein for subsequent docking simulations. To account for the ionization states of amino acid residues at a specific pH, the structures were initially subjected to protonation. Subsequently, the structures underwent refinement procedures aimed at eliminating all components other than the target protein. This involved the removal of superfluous chains and water molecules, as well as the assignment of partial charges to the remaining atoms. The process of energy minimization was employed to determine the protein conformations that exhibit the highest stability. The MMFF94X force field was utilized to represent the internal charge distribution. There were adjustments made to the docking technique to improve its reliability and accuracy. In this analysis, we made some minor alterations, such as using the Triangle Matcher placement method and the London dG score formula. To improve ligand binding prediction accuracy, a specialized procedure was developed. Careful analysis was performed to evaluate the binding affinity and interaction mode of the first co-crystallized ligand, and the results were compared to those published in prior research to verify the effectiveness of the improved methodology. This contrast was used as a benchmark against which the credibility of the docking findings could be assessed. After running the docking simulations, the resulting data was subjected to a thorough analysis. The selection of binding modes with high binding affinity was based on their potential to offer valuable insights into the intended targets, warranting further investigation. Quantitative assessments of the ligand-receptor interactions were conducted by estimating the docking scores and binding energies, utilizing the specified binding modes. The dependability and accuracy of the docking predictions were enhanced by the use of a thorough docking process that included the positioning of Triangle Matcher and the use of the London dG scoring function. High-affinity binding modes were identified after an evaluation of the ligand's binding interactions, allowing for a comprehensive analysis of the ligand's potential binding affinity and energetics. Fifty different protein conformations were created throughout our study. The interactions between the ligand (compound 5) and the amino acid residues were then studied by evaluating these conformations. In addition, the determination of the binding energy for each conformation was performed in order to gain a deeper understanding of the chemical's stability and potential affinity within the binding site. This was achieved by quantifying the strength of the interactions between the ligand and the protein.

## Conclusion

The presented study provides compelling evidence for the potential therapeutic value of a novel class of 8-methoxycoumarin-3-carboxamides in the treatment of hepatocellular carcinoma (HCC). Liver cancer remains a significant global health challenge, and existing pharmacological treatments have limitations, making the search for effective and safe therapeutic agents imperative. The 8-methoxycoumarin-3-carboxamides synthesized in this study demonstrated remarkable antiproliferative activity against HepG2 cells, a representative model of HCC. Notably, compound **5** emerged as the most promising candidate, displaying exceptional inhibitory effects with an IC_50_ value of 0.9 µM, surpassing the anticancer drug staurosporine while exhibiting minimal toxicity to normal cells. The mechanisms underlying the antiproliferative activity of compound **5** were elucidated, showing its ability to induce cell cycle arrest during the G1/S phase and trigger apoptosis without significant necrosis in HepG2 cells. Furthermore, compound **5** effectively activated caspase3/7 proteins and disrupted β-tubulin polymerization, indicating its potential to interfere with critical cellular processes involved in cancer cell growth and survival. The molecular modeling analysis reaffirmed the strong binding affinity of compound **5** to the active cavity of β-tubulin protein, further supporting its potential as an effective therapeutic agent targeting HCC. The presented findings underscore the promising therapeutic potential of 8-methoxycoumarin-3-carboxamides, especially compound **5**, in combatting hepatocellular carcinoma. Although these results are encouraging, further studies, including in vivo experiments, are warranted to validate the efficacy of compound **5** and related analogues. Nevertheless, our research lays the foundation for the discovery of new, potent anti-HCC agents and provides valuable insights into the mechanisms of action that can guide future drug development efforts.

### Supplementary Information

**Additional file 1: Scheme S1.** Main fragmentation pattern of compounds 4 and 6. **Scheme S1.** Main fragmentation pattern of compounds 5 and 7. **Figure S1a.** 1H NMR spectra of compound 2. **Figure S1b.** Mass spectrum of compound 2. **Figure S2a.** 1H NMR spectra of compound 3. **Figure S2b.** Mass spectrum of compound 3. **Figure S3a.** 1H NMR and 13C NMR spectra of compound 4. **Figure S3b.** Mass spectrum of compound 4. **Figure S4a.** 1H NMR and 13C NMR spectra of compound 5. **Figure S4b.** Mass spectrum of compound 5. **Figure S5.** Mass spectrum of compound 6. **Figure S6a.** 1H NMR and 13C NMR spectra of compound 7. **Figure S6b.** Mass spectrum of compound 7. **Figure S7a.** 1H NMR and 13C NMR spectra of compound 8. **Figure S7b.** Mass spectrum of compound 8. **Figure S8a.** 1H NMR and 13C NMR spectra of compound 9. **Figure S8b.** Mass spectrum of compound 9. **Figure S9.** The dose-dependent cytotoxic activity of synthesized compound 5 toward WI 38 cells, as compared to STU. The presented data shows the mean ± standard deviation of the mean obtained from at least three independent experiments. **Table S1.** Cytotoxic evaluation of compounds 4–9, as compared to STU, against human liver carcinoma HepG2.

## Data Availability

All data generated or analyzed during this study are included in this article (and its additional information files). Samples from compounds 4–9 are available from the authors.
