# Supplementary material for: Novel 8-Methoxycoumarin-3-Carboxamides with potent anticancer activity against liver cancer via targeting caspase-3/7 and β-tubulin polymerization
Source: BMC Chem. 2023 Dec 2;17(1):174. doi: 10.1186/s13065-023-01063-5 (PMC10693084; doi:10.1186/s13065-023-01063-5)
Supplement: Supplementary file 1 — Additional file 1: Scheme S1. Main fragmentation pattern of compounds 4 and 6. Scheme S1. Main fragmentation pattern of compounds 5 and 7. Figure S1a. 1H NMR spectra of compound 2. Figure S1b. Mass spectrum of compound 2. Figure S2a. 1H NMR spectra of compound 3. Figure S2b. Mass spectrum of compound 3. Figure S3a. 1H NMR and 13C NMR spectra of compound 4. Figure S3b. Mass spectrum of compound 4. Figure S4a. 1H NMR and 13C NMR spectra of compound 5. Figure S4b. Mass spectrum of compound 5. Figure S5. Mass spectrum of compound 6. Figure S6a. 1H NMR and 13C NMR spectra of compound 7. Figure S6b. Mass spectrum of compound 7. Figure S7a. 1H NMR and 13C NMR spectra of compound 8. Figure S7b. Mass spectrum of compound 8. Figure S8a. 1H NMR and 13C NMR spectra of compound 9. Figure S8b. Mass spectrum of compound 9. Figure S9. The dose-dependent cytotoxic activity of synthesized compound 5 toward WI 38 cells, as compared to STU. The presented data shows the mean ± standard deviation of the mean obtained from at least three independent experiments. Table S1. Cytotoxic evaluation of compounds 4–9, as compared to STU, against human liver carcinoma HepG2. [file 13065_2023_1063_MOESM1_ESM.pdf]

Supplementary Materials

Novel 8-Methoxycoumarin-3-Carboxamides with potent Anticancer activity against Liver Cancer *via* targeting caspase-3/7 and β-tubulin polymerization

Ahmad Alzamami [1], Eman M. Radwan [2], Eman Abo-Elabass [3], Mohammed El Behery [3], Hussah Abdullah Alshwyeh [4,5], Ebtesam Al-Olayan[6], Abdulmalik S. Altamimi [7], Nashwah G M Attallah [8], Najla Altwaijry [9], Mariusz Jaremko [10,*], and Essa M. Saied [11,12,*]

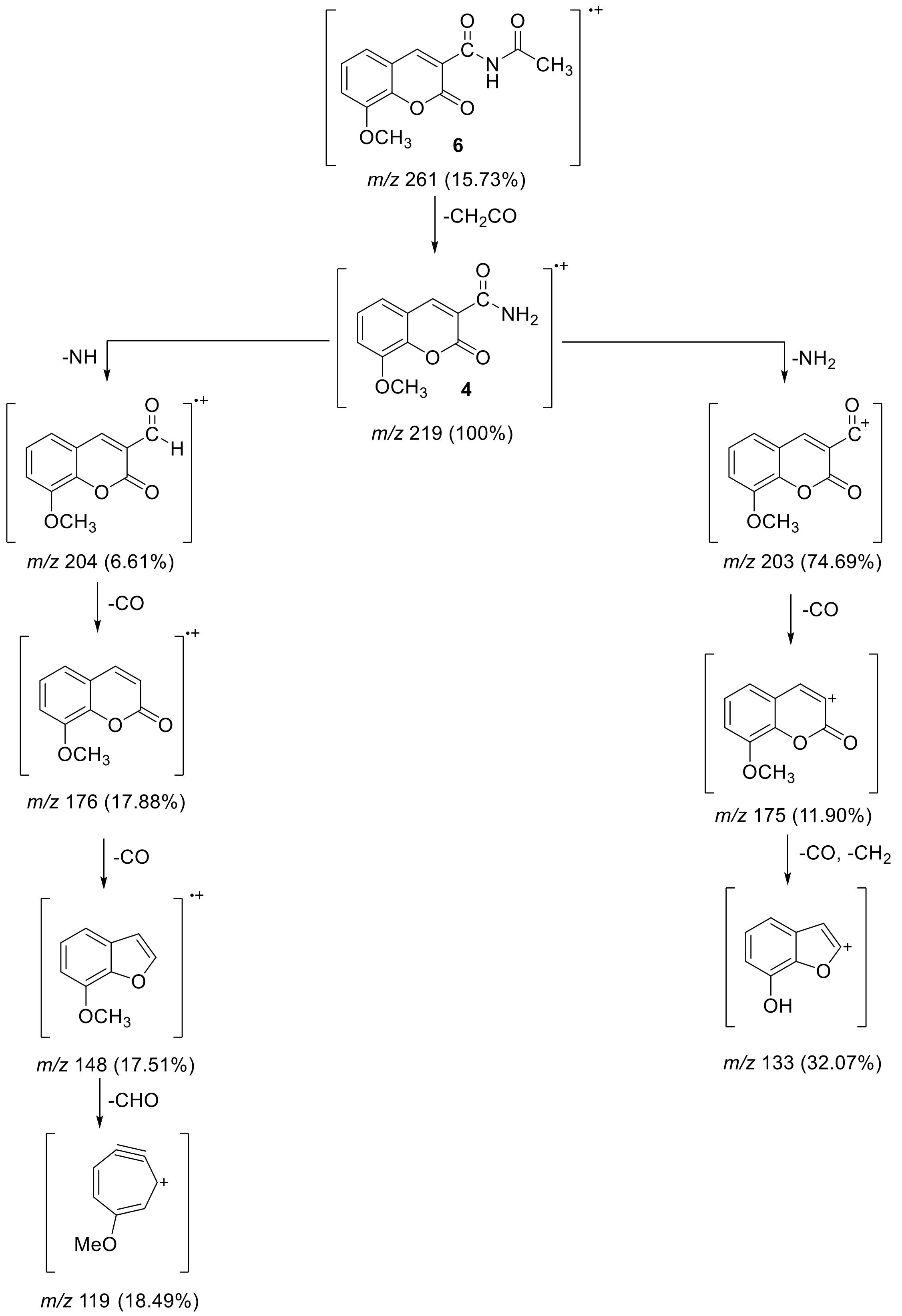

Scheme S1: Main fragmentation pattern of compounds 4 and 6.

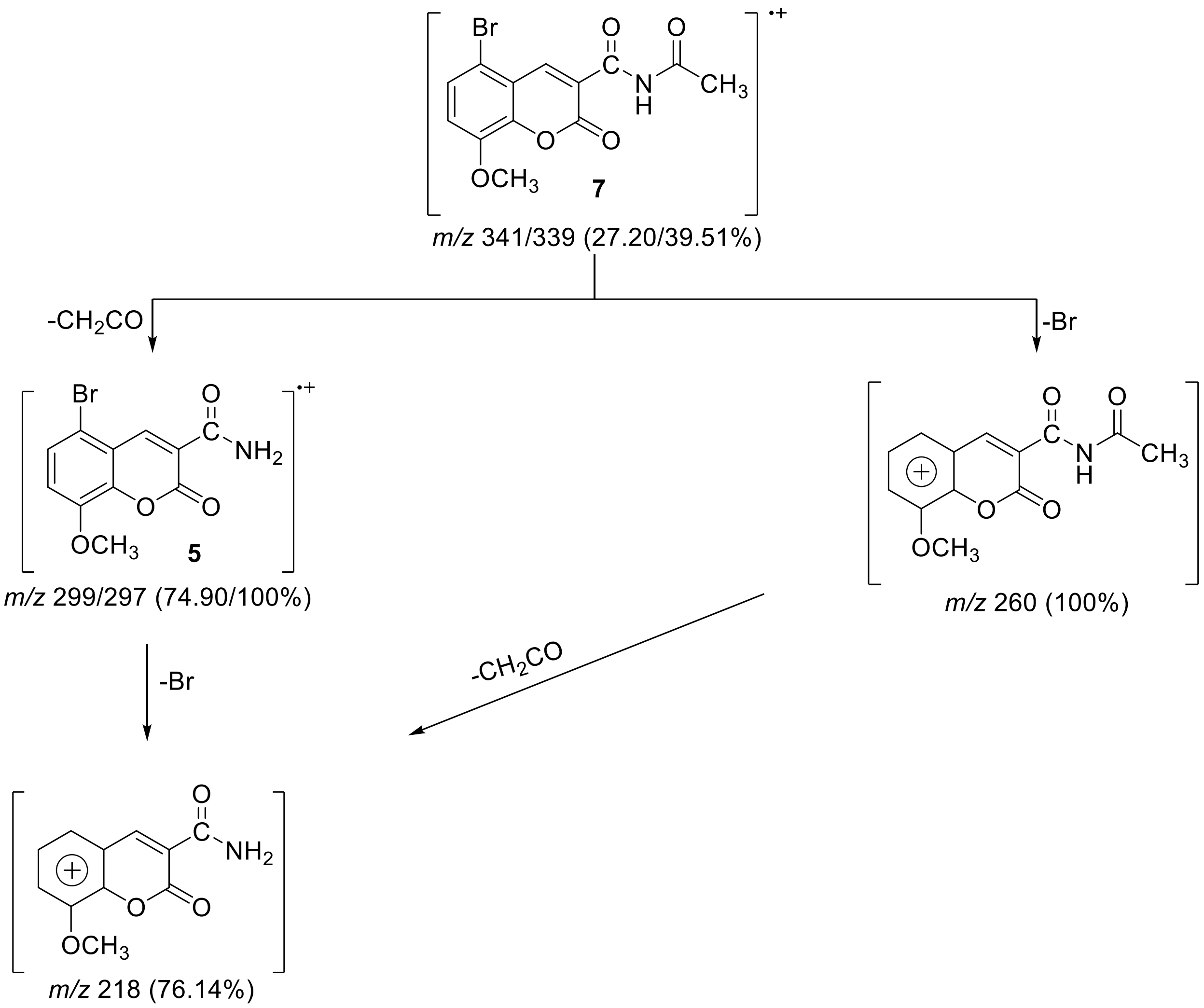

Scheme S1: Main fragmentation pattern of compounds 5 and 7.

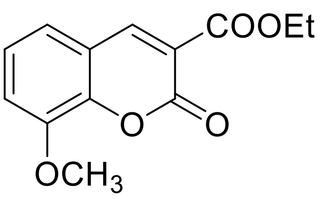

2

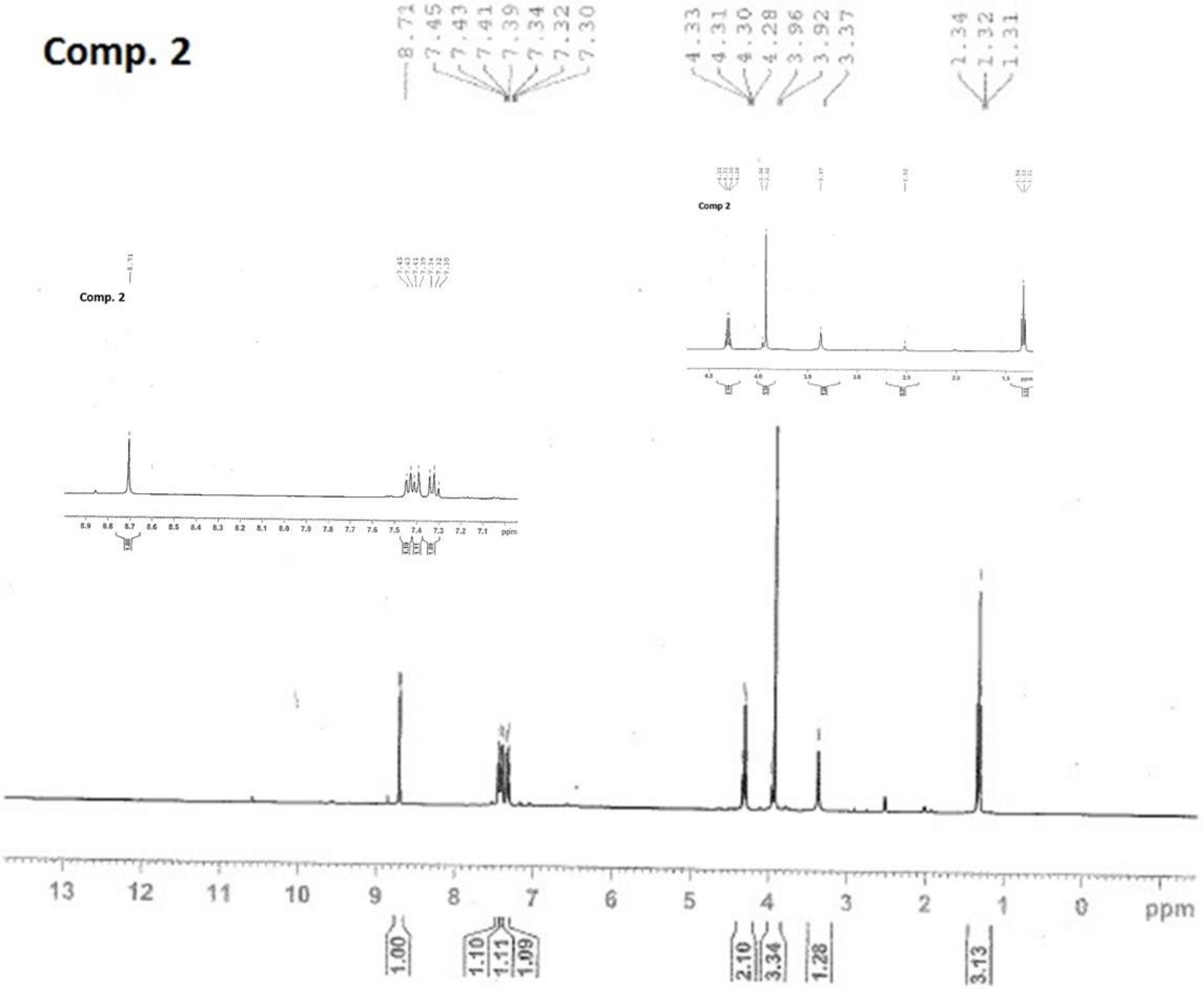

Figure S1a. ^{1}H NMR spectra of compound 2

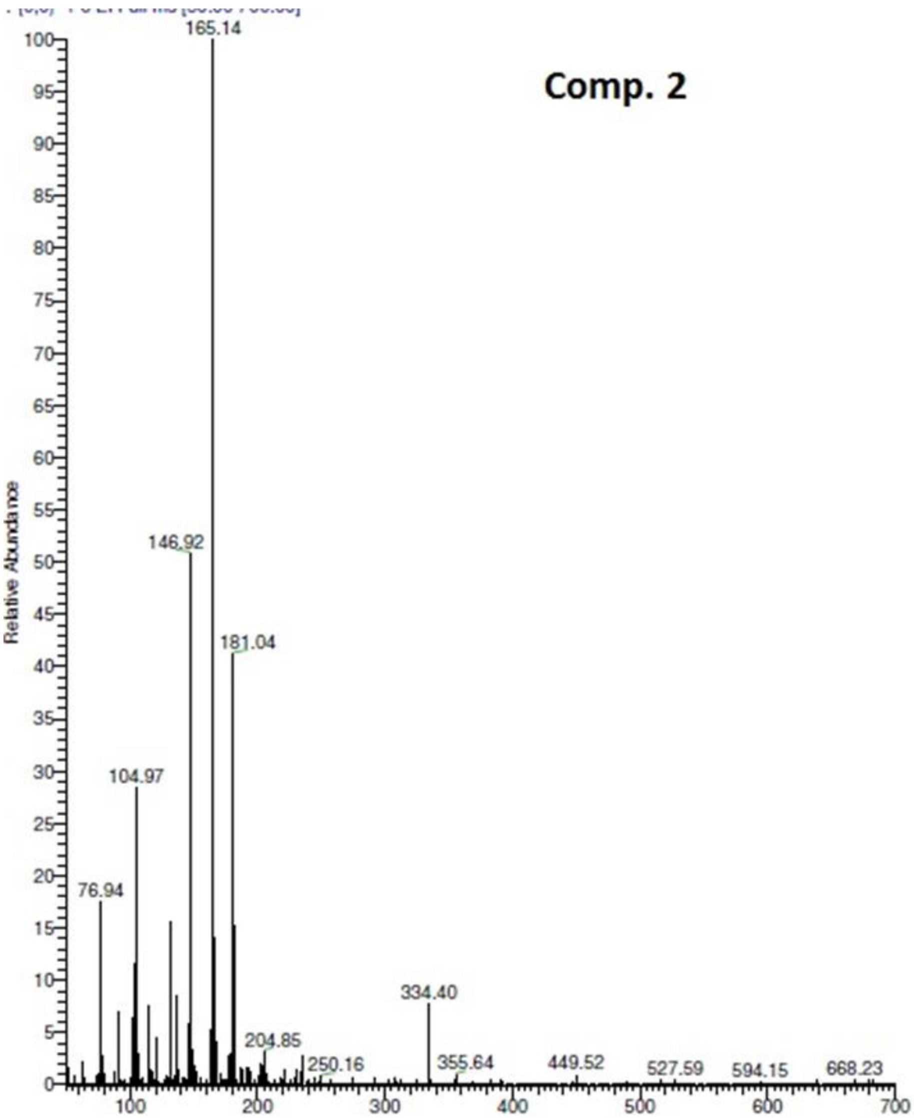

Figure S1b. Mass spectrum of compound 2.

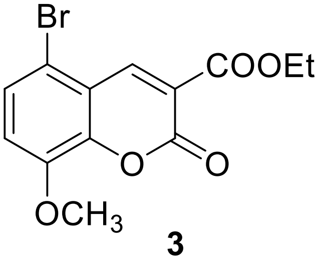

3

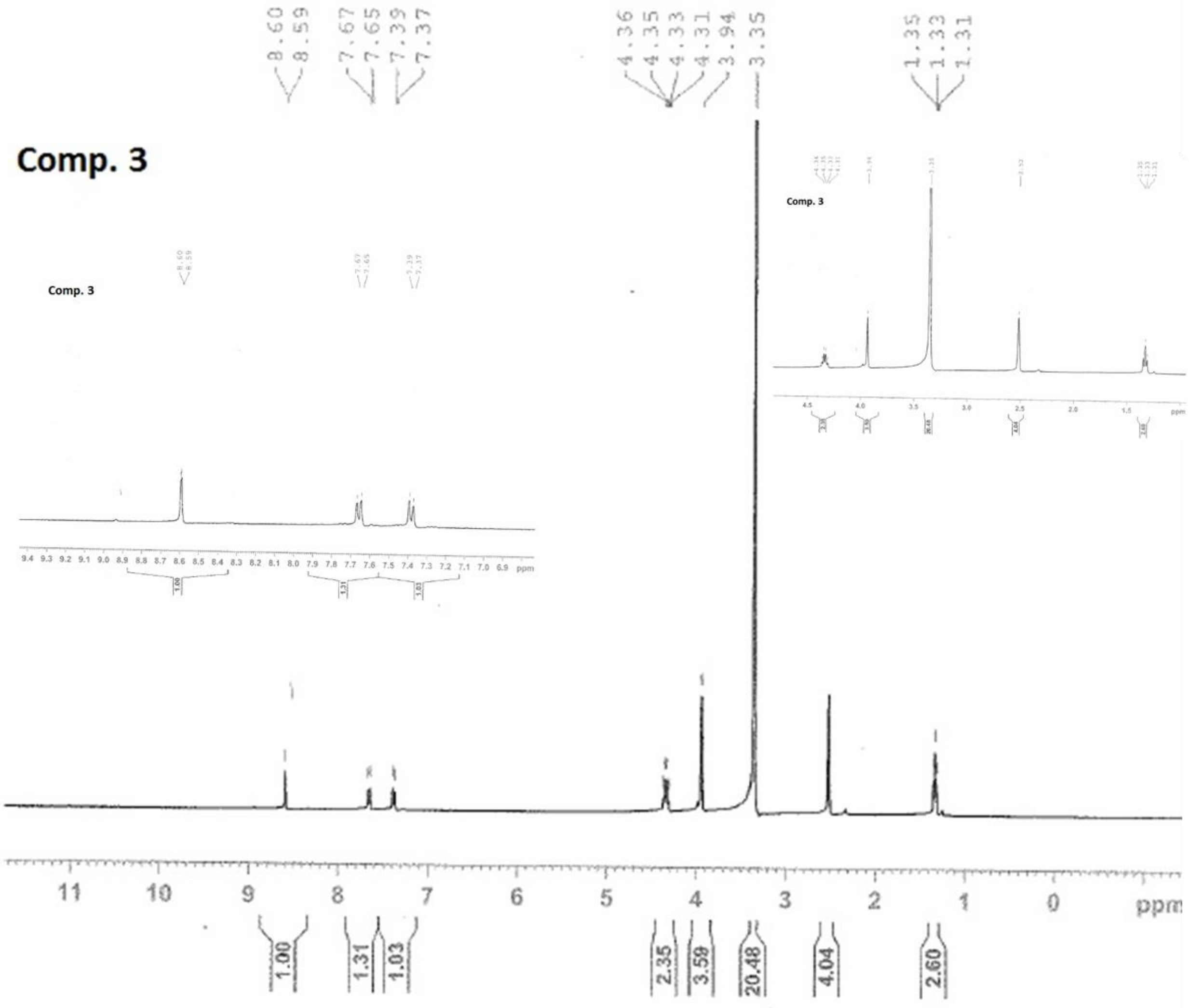

Figure S2a. ^{1}H NMR spectra of compound 3

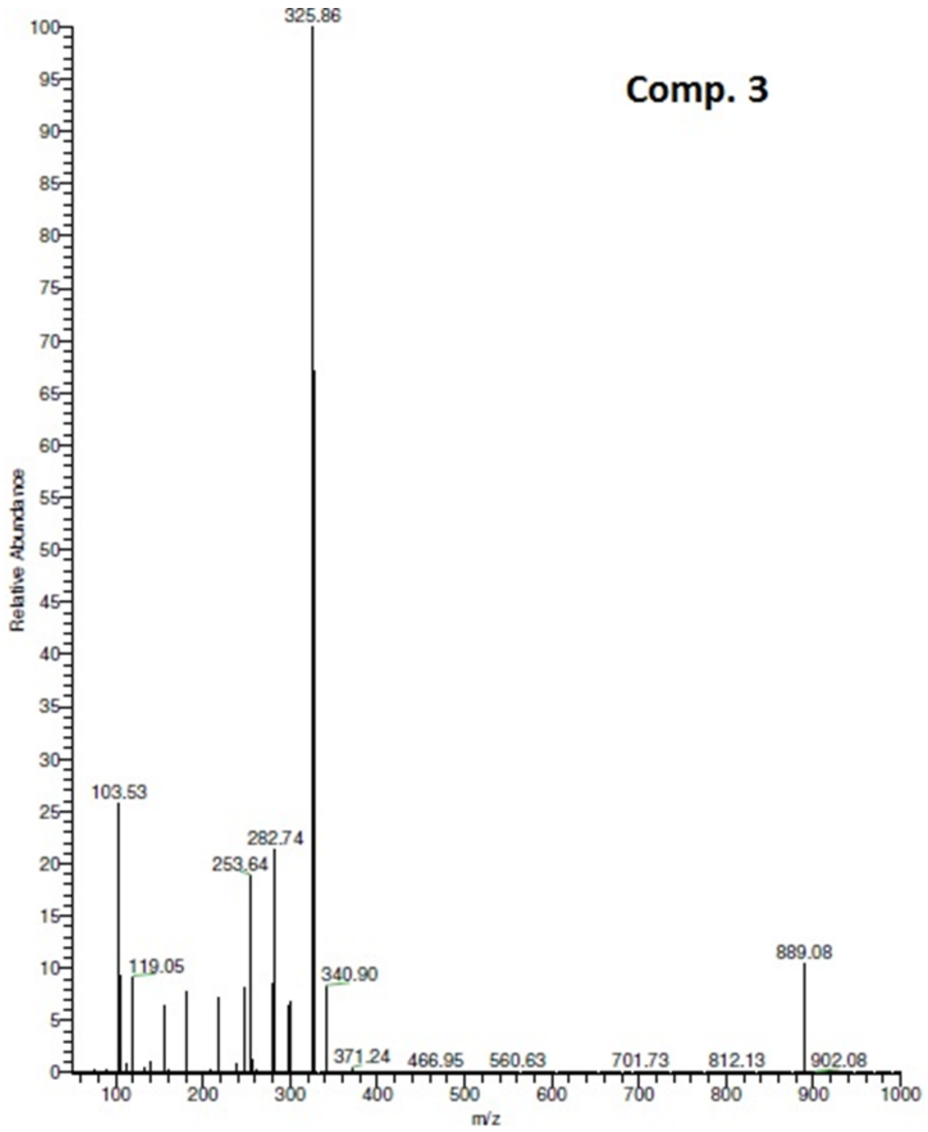

Figure S2b. Mass spectrum of compound 3.

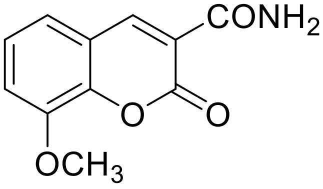
CONH2
O
O
OCH3

4

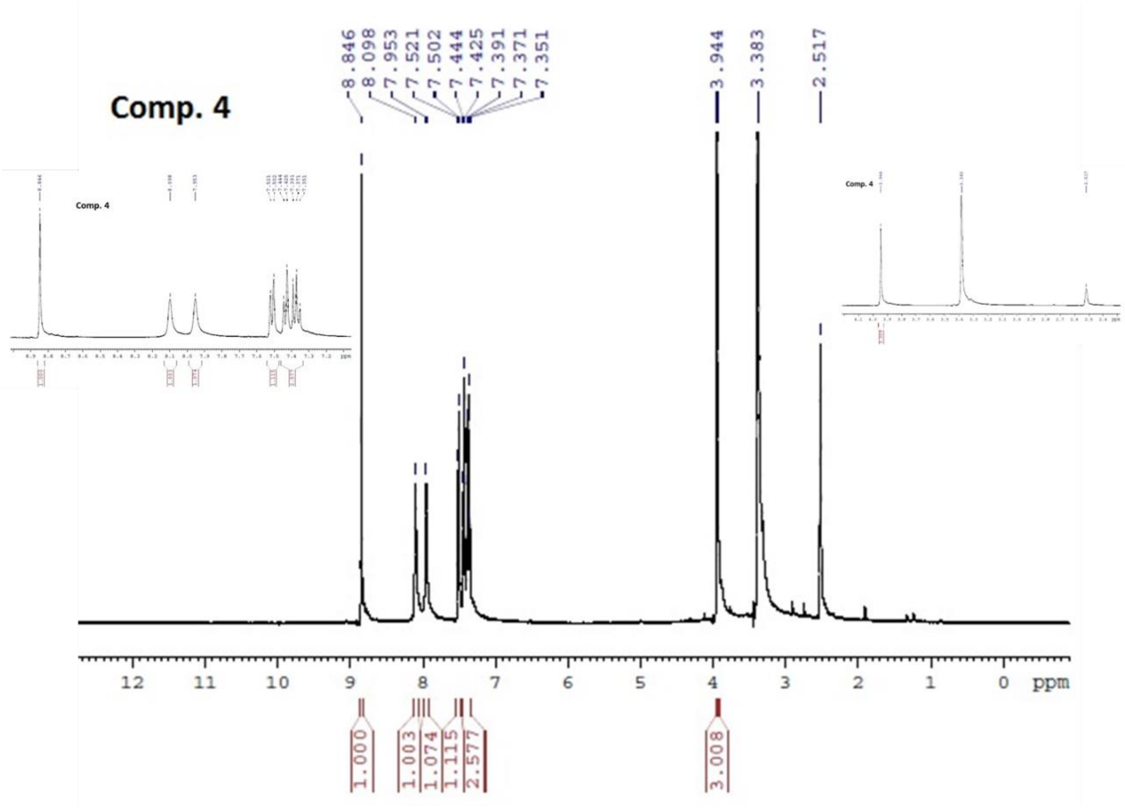
Comp. 4
8.846
8.098
7.953
7.521
7.502
7.444
7.425
7.391
7.371
7.351
3.944
3.383
2.517
12 11 10 9 8 7 6 5 4 3 2 1 0 ppm
1.000
1.003
1.074
1.115
2.577
3.008

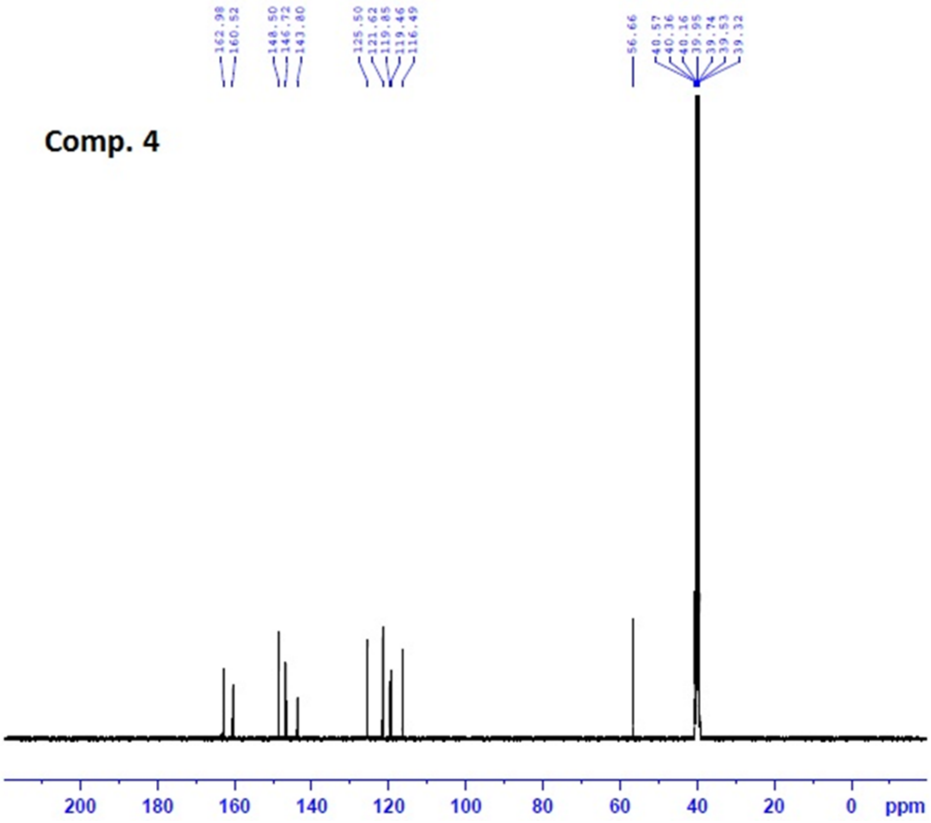

Figure S3a. ^{1}H **NMR and** ^{13}C **NMR spectra of compound 4**

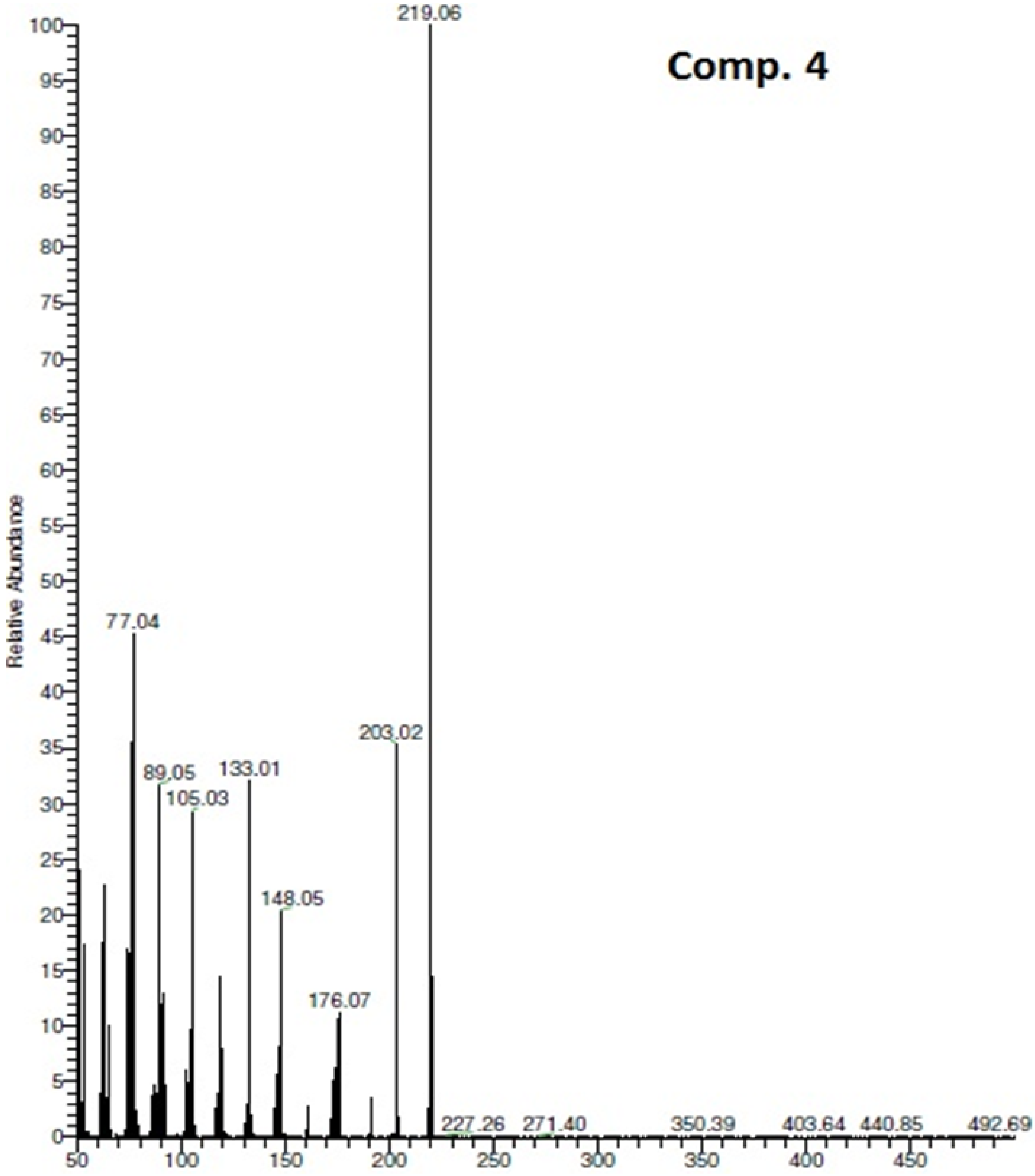

Figure S3b. Mass spectrum of compound 4.

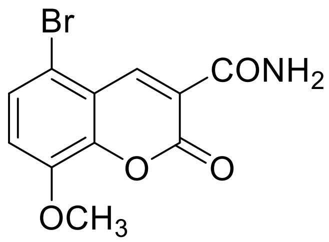
Br
CONH2
O
O
OCH3

5

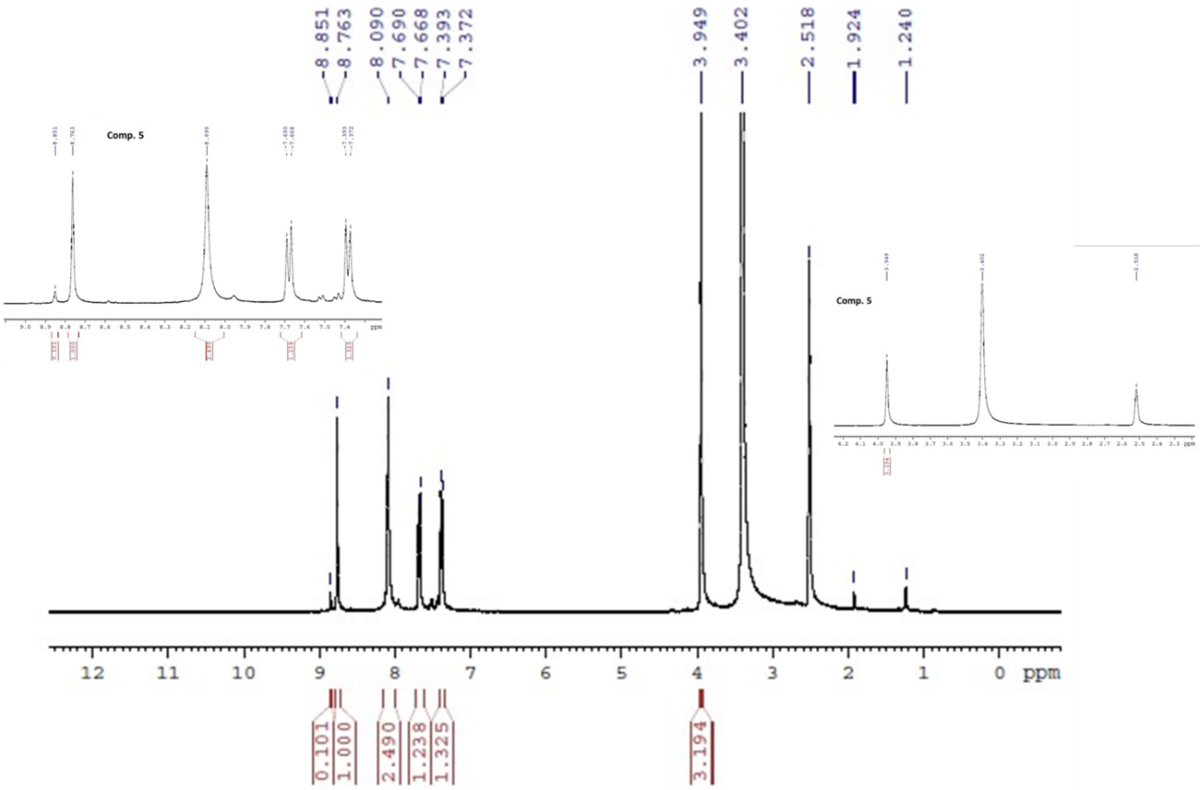
8.851
8.763
8.090
7.690
7.668
7.393
7.372
3.949
3.402
2.518
1.924
1.240
Comp. 5
Comp. 5
12 11 10 9 8 7 6 5 4 3 2 1 0 ppm
0.101
1.000
2.490
1.238
1.325
3.194

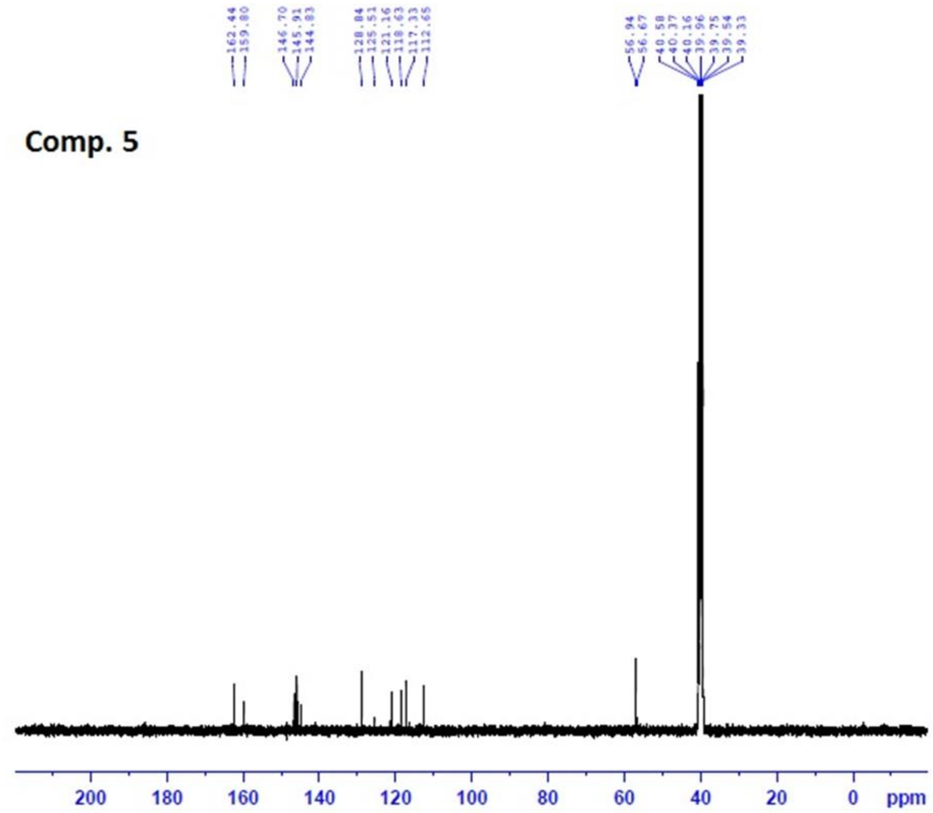

Figure S4a. ^{1}H NMR and ^{13}C NMR spectra of compound 5

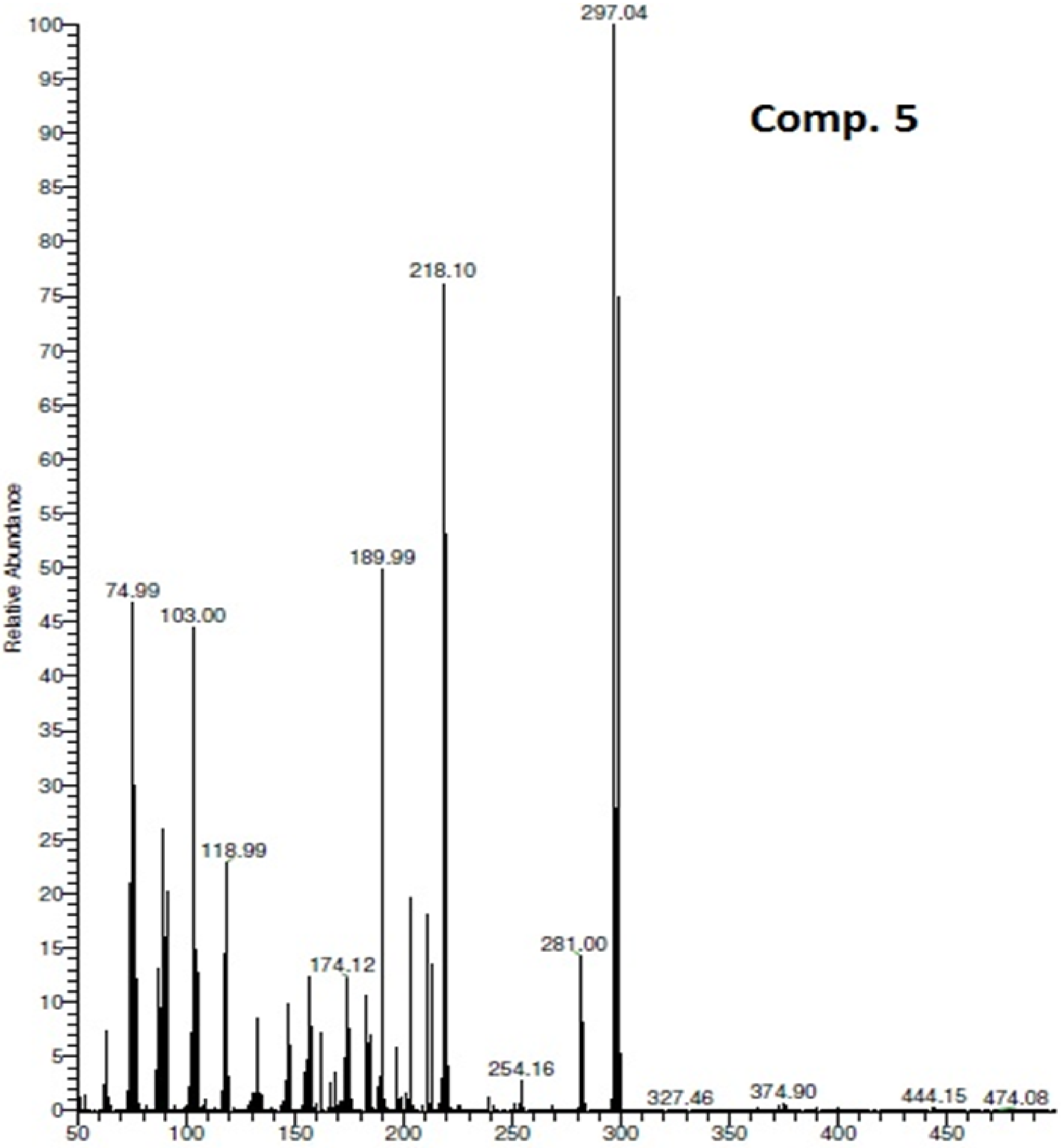

Figure S4b. Mass spectrum of compound 5.

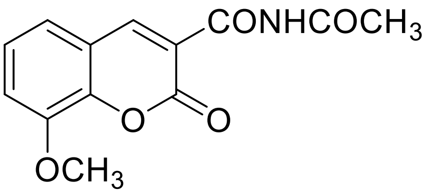

6

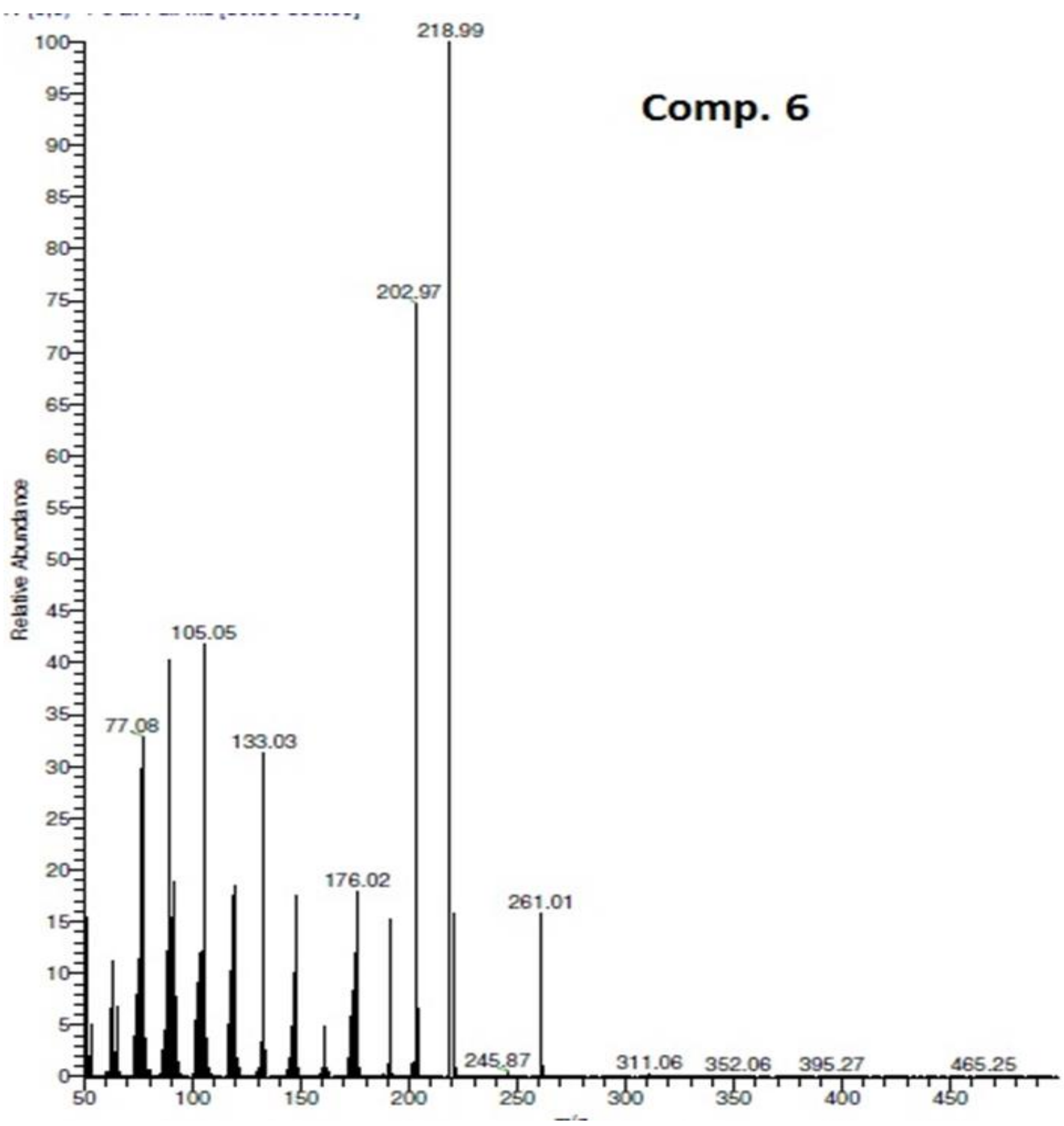

Figure S5. Mass spectrum of compound 6.

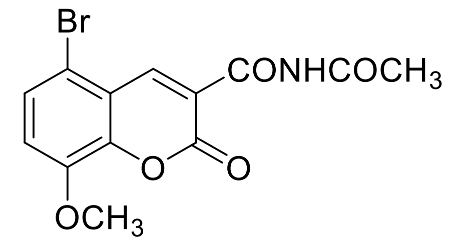
Br
CONHCOCH$_3$
O
O
OCH$_3$

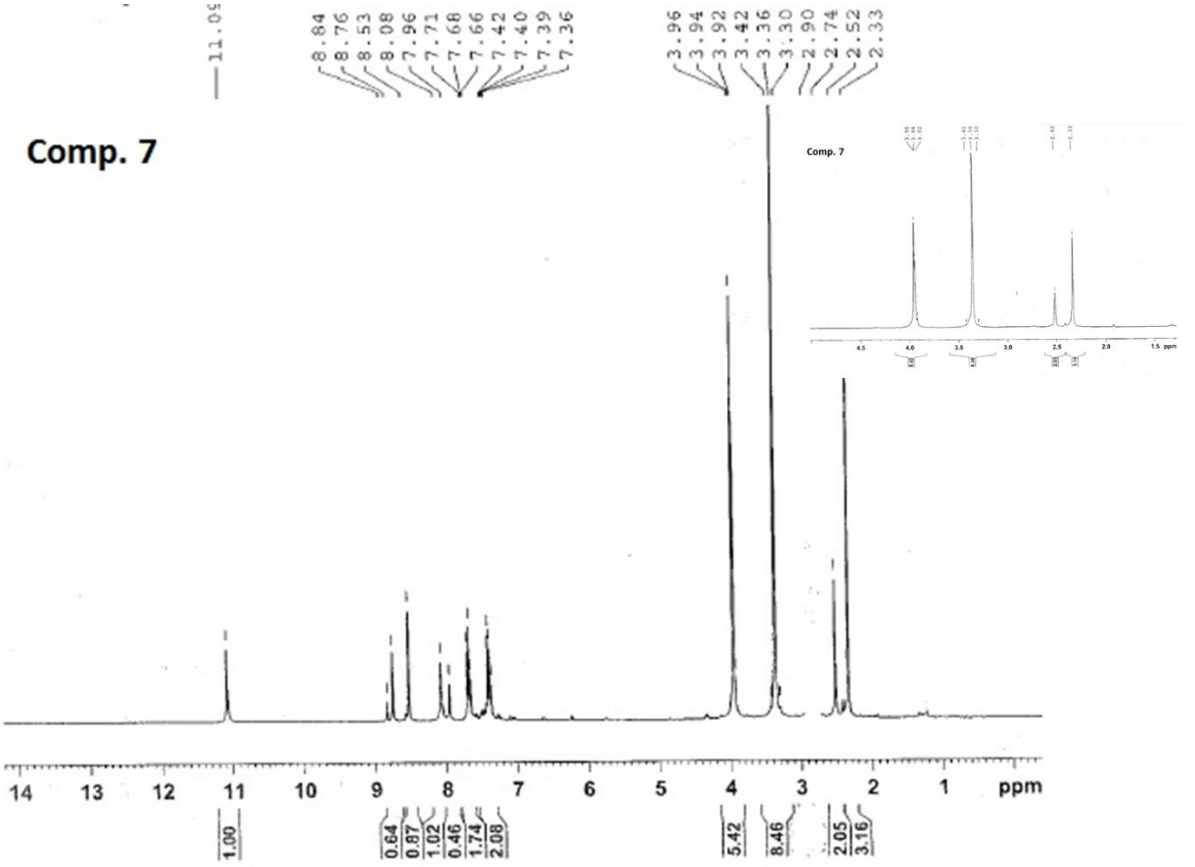
Comp. 7
11.09
8.84
8.76
8.53
8.08
7.96
7.71
7.68
7.66
7.42
7.40
7.39
7.36
3.96
3.94
3.92
3.42
3.36
3.30
2.90
2.74
2.52
2.33
ppm
1.00
0.64
0.87
1.02
0.46
1.74
2.08
5.42
8.46
2.05
3.16

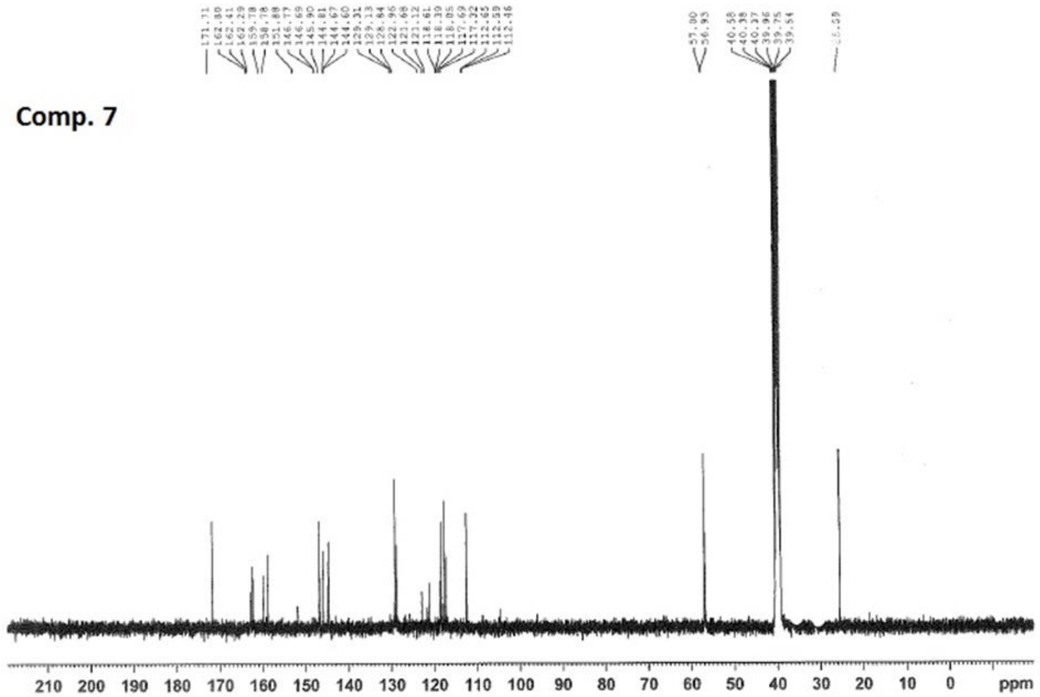

Figure S6a. ^{1}H NMR and ^{13}C NMR spectra of compound 7.

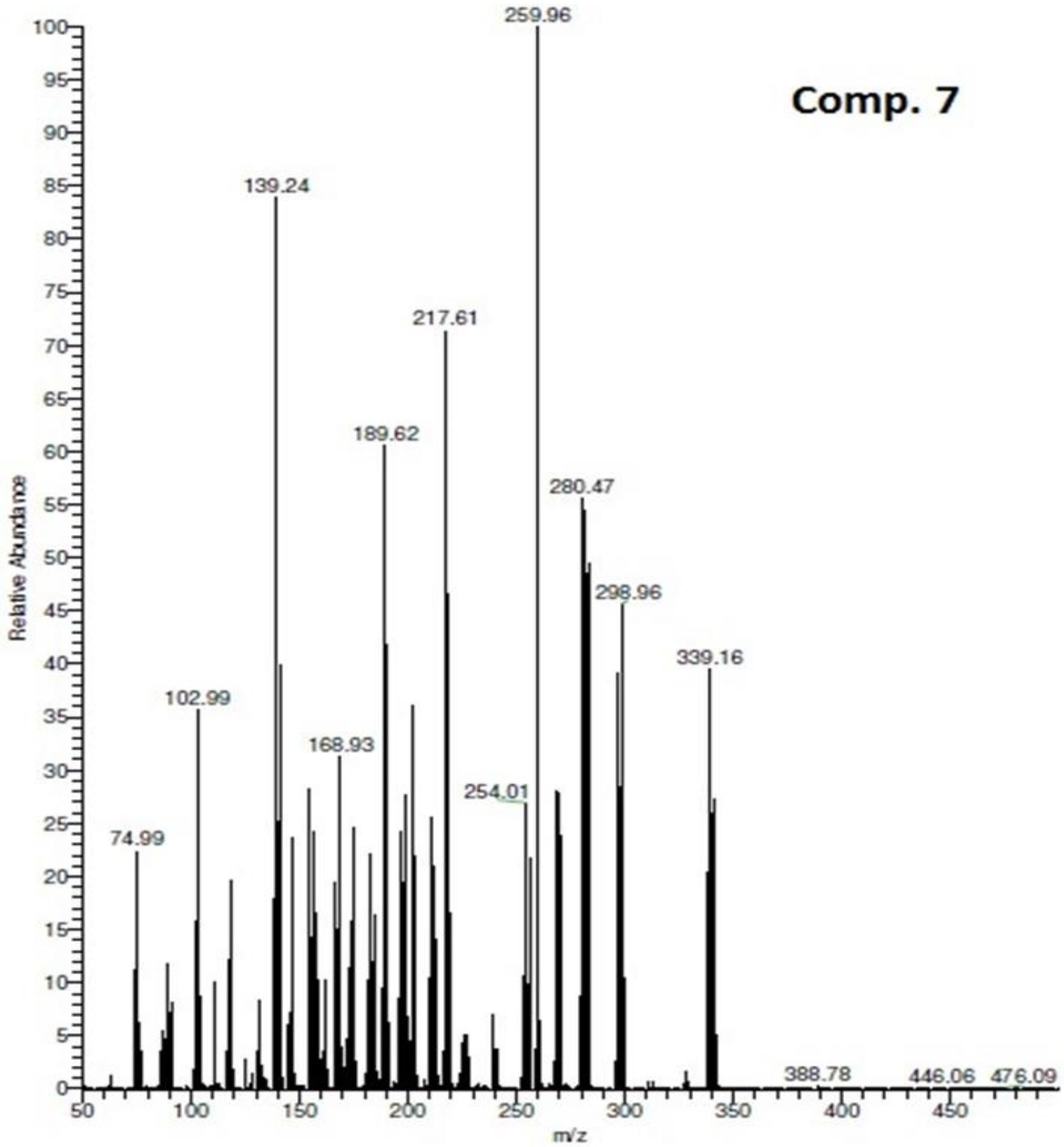

Figure S6b. Mass spectrum of compound 7.

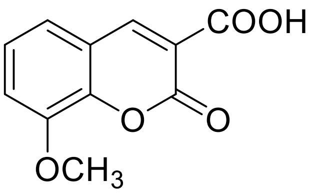
COOH
O
O
OCH3

8

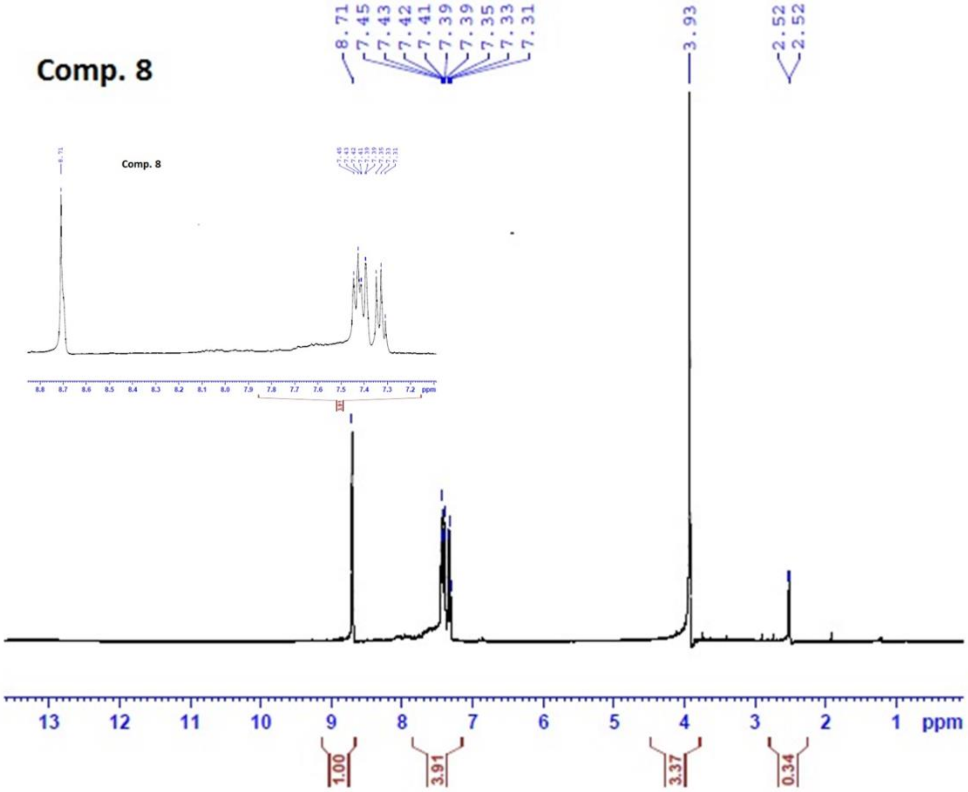
Comp. 8
8.71
7.45
7.43
7.42
7.41
7.39
7.39
7.35
7.33
7.31
3.93
2.52
2.52
13 12 11 10 9 8 7 6 5 4 3 2 1 ppm
1.00
3.91
3.37
0.34

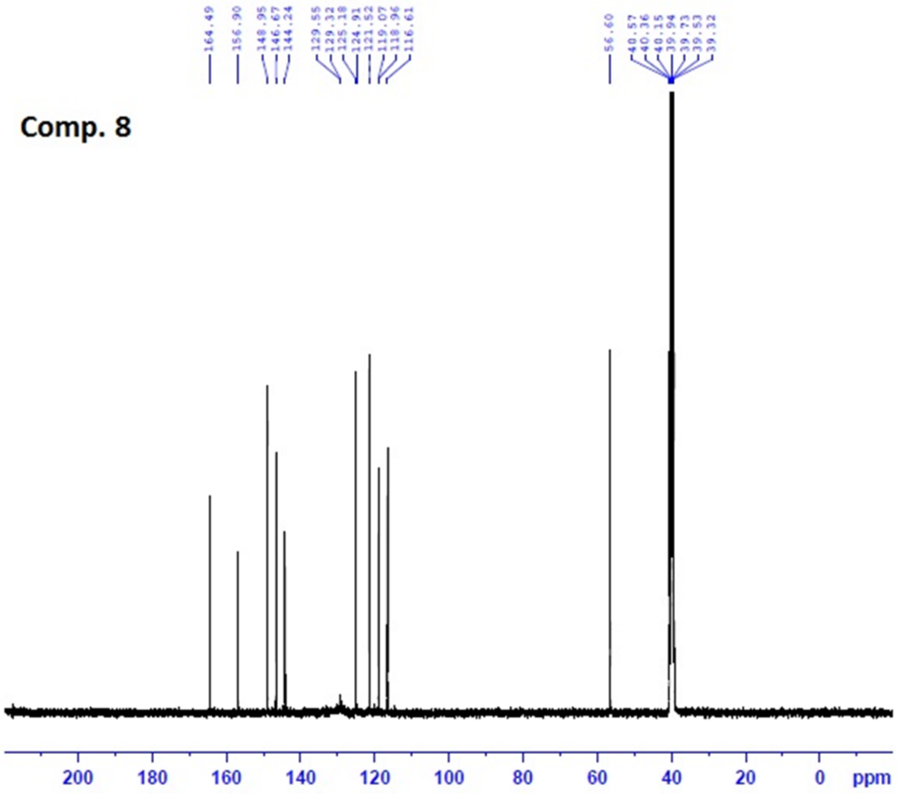

Figure S7a. ^{1}H NMR and ^{13}C NMR spectra of compound 8.

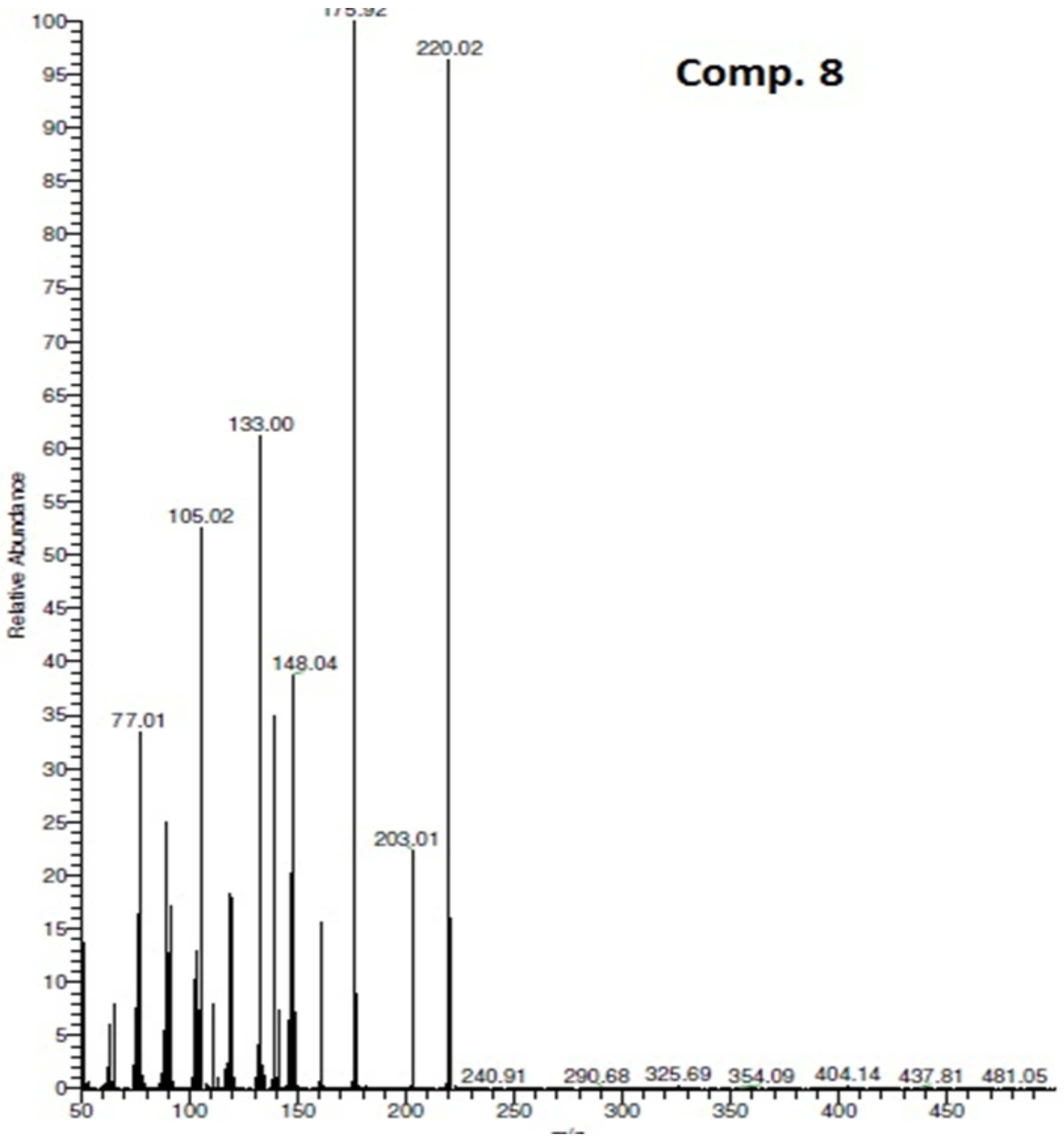

Figure S7b. Mass spectrum of compound 8.

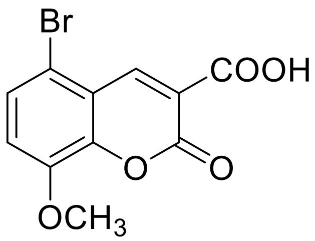

9

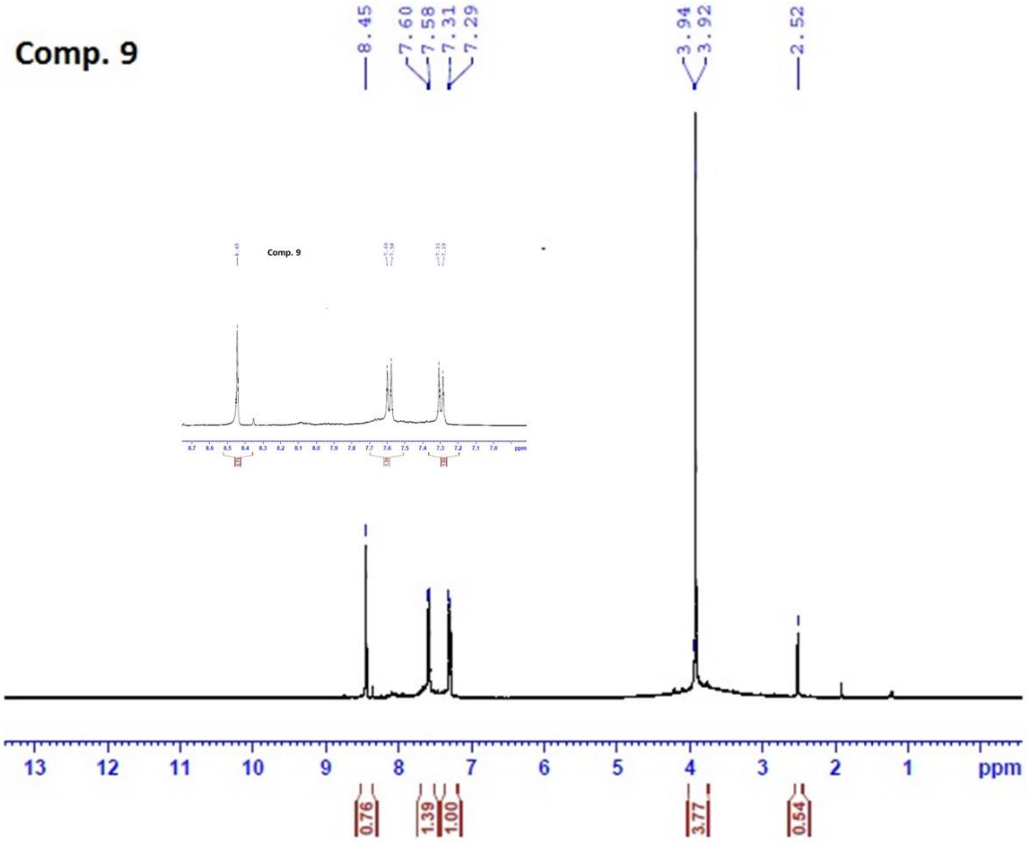
Comp. 9
8.45
7.60
7.58
7.31
7.29
3.94
3.92
2.52
13
12
11
10
9
8
7
6
5
4
3
2
1
ppm
0.76
1.39
1.00
3.77
0.54

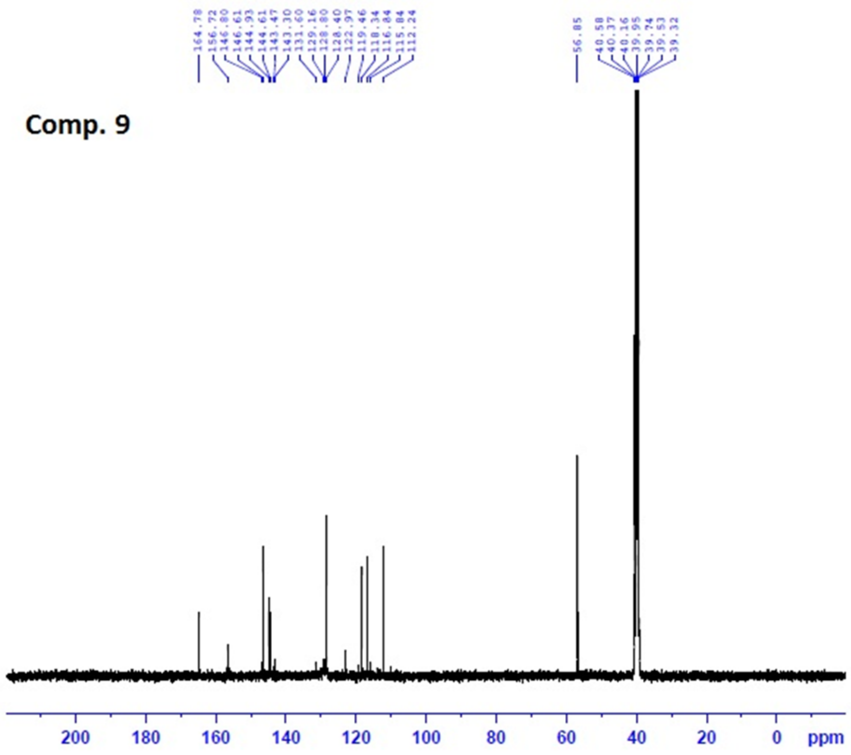

Figure S8a. ^{1}H NMR and ^{13}C NMR spectra of compound 9.

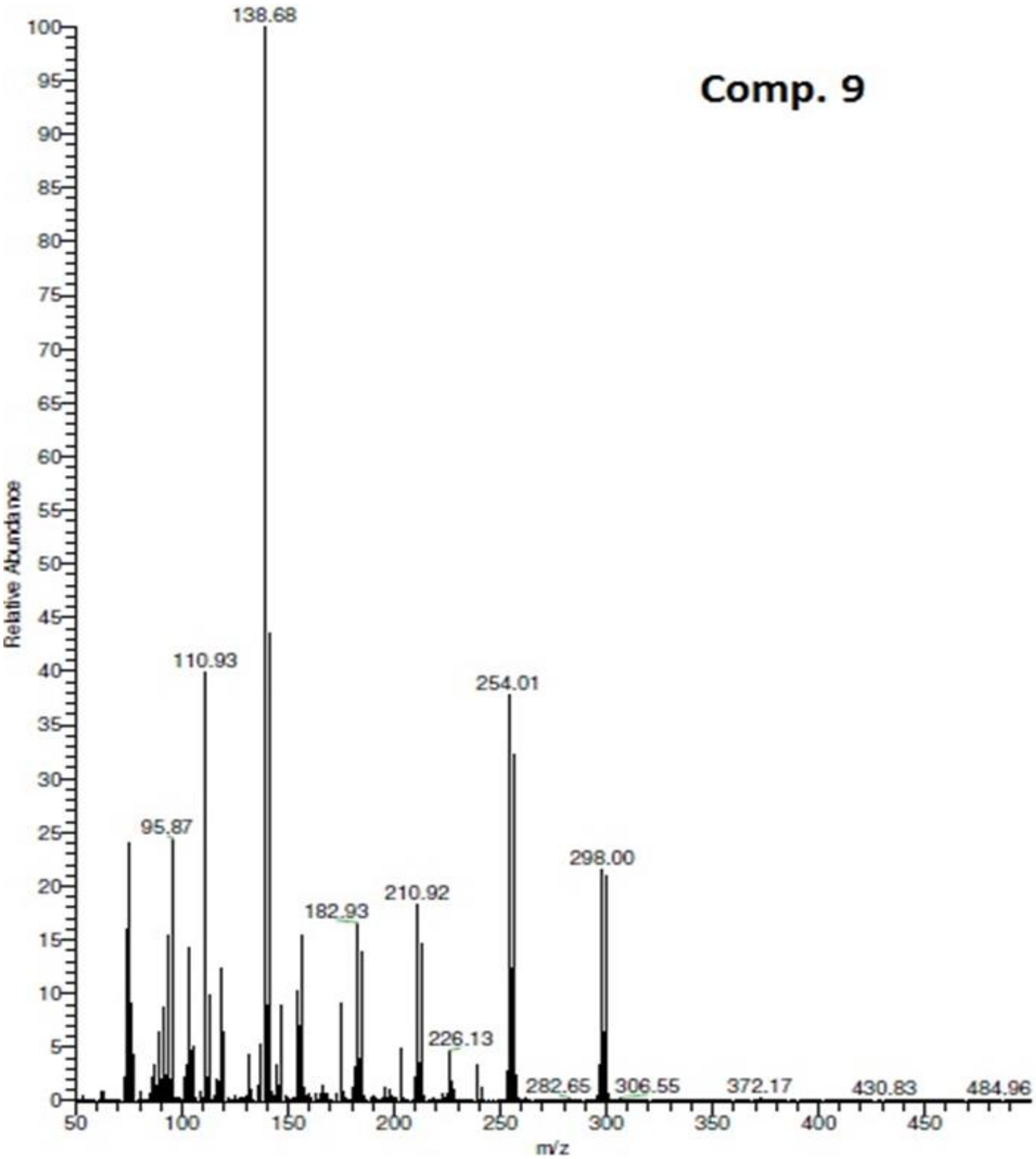

Figure S8b. Mass spectrum of compound 9.

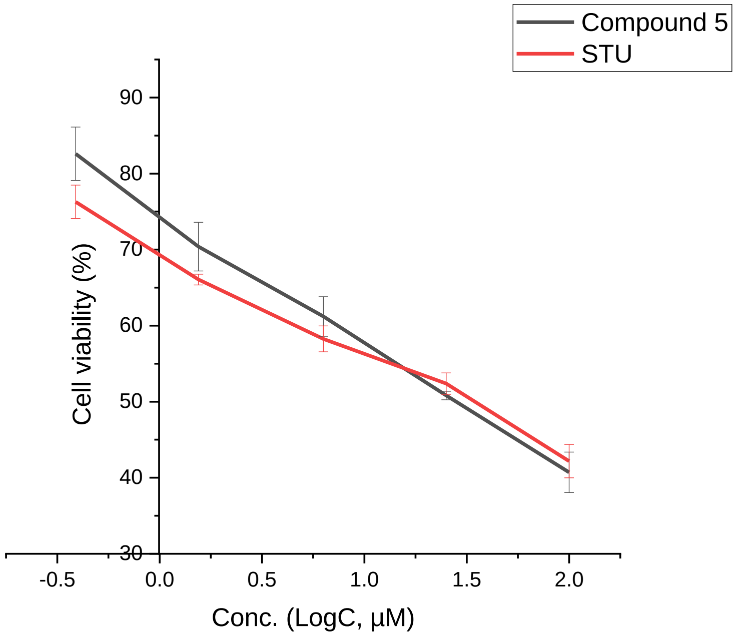

Figure S9. The dose-dependent cytotoxic activity of synthesized compound **5** toward WI 38 cells, as compared to STU. The presented data shows the mean ± standard deviation of the mean obtained from at least three independent experiments.

Table S1: Cytotoxic evaluation of compounds **4-9**, as compared to STU, against human liver carcinoma HepG2.

**Concentration**	**HepG2-Cell Viability (%)**						
**LogC, μM**	**Compound 4**	**Compound 5**	**Compound 6**	**Compound 7**	**Compound 8**	**Compound 9**	**STU**
**2**	37.94±2.75	34.02±3.21	27.04±2.87	45.03±3.1	24.59±1.84	42.17±2.94	29.43±3.14
**1.4**	47.29±3.09	43.91±2.73	37.53±2.14	53.19±2.87	35.26±1.93	50.19±3.07	39.57±2.71
**0.8**	57.78±2.46	52.05±2.11	44.64±1.98	60.52±2.64	40.63±1.66	55.34±2.24	55.04±1.96
**0.19**	65.8±1.73	60.49±1.84	54.47±1.72	68.52±2.36	46.68±1.21	66.86±1.86	62.49±1.62
**-0.41**	75.81±1.48	68.4±1.52	59.86±1.33	77.18±1.73	53.85±1.34	73.23±1.47	75.85±1.26
